# Conjugated porphyrin polymer films with nickel single sites for the electrocatalytic oxygen evolution reaction†

**DOI:** 10.1039/d2ta07748e

**Published:** 2023-01-26

**Authors:** Deepak Bansal, Drialys Cardenas-Morcoso, Nicolas Boscher

**Affiliations:** a Materials Research and Technology Department, Luxembourg Institute of Science and Technology 28 Avenue des Hauts-Fourneaux Esch-Sur-Alzette Luxembourg Deepak.bansal@list.lu Nicolas.boscher@list.lu

## Abstract

Directly fused nickel(ii) porphyrins are successfully investigated as heterogeneous single-site catalysts for the oxygen evolution reaction (OER). Conjugated polymer thin films from Ni(ii) 5,15-(di-4-methoxycarbonylphenyl)porphyrin (pNiDCOOMePP) and Ni(ii) 5,15-diphenylporphyrin (pNiDPP) showed an OER onset overpotential of 270 mV, and current densities of 1.6 mA cm^−2^ and 1.2 mA cm^−2^ at 1.6 V *vs.* RHE, respectively, representing almost a hundred times higher activity than those of monomeric thin films. The fused porphyrin thin films are more kinetically and thermodynamically active than their non-polymerized counterparts mainly due to the formation of conjugated structures enabling a dinuclear radical oxo-coupling (ROC) mechanism at low overpotential. More importantly, we have deciphered the role of the porphyrin substituent in the conformation and performance of porphyrin conjugated polymers as (1) to control the extension of the conjugated system during the oCVD reaction, allowing the retention of the valence band deep enough to provide a high thermodynamic water oxidation potential, (2) to provide a flexible molecular geometry to facilitate O_2_ formation from the interaction between the Ni–O sites and to weaken the π-bond of the *Ni–O sites for enhanced radical character, and (3) to optimize the water interaction with the central metal cation of the porphyrin for superior electrocatalytic properties. These findings open the scope for molecular engineering and further integration of directly fused porphyrin-based conjugated polymers as efficient heterogeneous catalysts.

## Abstract 2

Nickel(ii) porphyrins are polymerized *via* chemical vapor deposition into highly conjugated structures. The careful selection of the substituent enables the OER to be performed at low overpotential.

## Graphical abstract

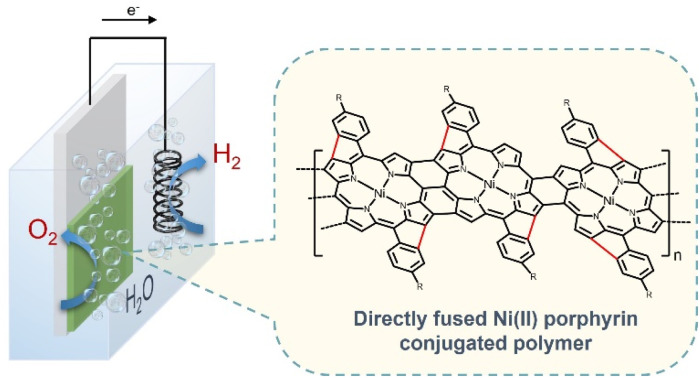

## Introduction

The development of oxygen evolution reaction (OER) catalysts with low overpotentials is an essential requirement for the implementation of sustainable fuel production systems with low energy costs. In this context, the operating mechanism has a significant effect on the theoretically achievable minimal overpotential. The prevalent mechanism proposed for molecular single-site catalysts is the water nucleophilic attack (WNA) pathway.^1^ However, catalysts operating *via* WNA have a minimum fundamental overpotential of 300 mV due to a scaling relationship.^2–4^ Such an intrinsic limitation does not arise for catalysts that operate *via* a radical oxo-coupling (ROC) mechanism, which should be therefore able to operate at lower overpotentials.^4,5^ The rational design and optimization of catalysts working *via* a ROC mechanism require fulfilling two important pre-requisites: (1) sufficient spin density on the oxygen atom of the metal-oxo species and (2) the proximity of two metal-oxo species to react and form an O–O bond.^4^ Therefore, meeting these conditions must be considered in the development of new OER electrocatalytic systems operating at lower overpotentials.

Metalloporphyrins have been actively investigated towards their potential applications as sensors,^6,7^ photosensitizers for solar cells,^8^ and catalysts.^9–11^ Inspired by their role in natural photosynthesis, porphyrin-based materials have notably been studied towards photo- and electro-catalytic water splitting reactions.^12–15^ The multiple applications of porphyrins are envisaged by their redox-active nature^16^ and interesting optoelectronic properties.^17–21^ Importantly, the optoelectronic properties of metalloporphyrins can be tuned by the incorporation of substituents into one or several of the eight β- and four *meso*-positions of the porphyrin ring,^22–24^ and/or from the introduction of a cation inside the porphyrin core.^25^ Therefore, judicious selection of a metalloporphyrin containing appropriate catalytic active metal centre and bearing pertinent substituents offers immense scope towards artificial light harvesting materials having potential application in electrocatalysis and photo-electrochemical processes.

Metalloporphyrins with different central metal cations such as Co,^26–28^ Mn,^29–32^ Fe^33,34^ and fewer Ni^35–39^ have been actively investigated as homogeneous water oxidation catalysts. However, weak interactions between catalysts and electrodes, low catalytic turnover frequency and difficulty in catalyst recycling are observed as the major limiting factors.^40–42^ Therefore, heterogenization, which significantly reduces the electrode-catalyst distance in comparison to homogeneous catalysis, is highly desirable. Heterogenization results in superior chemical stability and higher operational convenience for industrial applications.^40,42^ Yet, it is essential to ensure the efficient transport of electrons to the catalytic centres to promote high current densities.^43^ In this context, several strategies have been exploited to develop metalloporphyrin-based materials with improved optoelectronic and catalytic properties, including the immobilization of metalloporphyrins onto different conductive materials such as carbon nanotubes (CNTs),^39,44,45^ graphene oxide (GO)^46,47^ and indium or fluorine-doped tin oxide (ITO/FTO).^30,48^

The polymerization of metalloporphyrins is also a convenient approach to enhance their photo-electrochemical properties.^39,49^ Particularly, the direct fusion of metalloporphyrins into highly conjugated oligomers/polymers is an effective way to improve their optoelectronic and catalytic properties.^50–52^ The direct fusion of multiple porphyrin units considerably reduces their HOMO–LUMO band gap leading to a drastic shift of absorption towards the NIR region.^53^ In addition, directly fused metalloporphyrins exhibit significantly enhanced electronic properties, such as conductivity, owing to long range electronic conjugation.^53–56^ However, despite their attractive electronic, electrochemical, and optical features, conjugated polymers based on directly fused porphyrins struggle to meet the requirements of practical applications due to their very weak solubility and non-meltability.

Recently, our group reported the first straightforward route to the preparation, engineering and deposition of directly fused porphyrin conjugated polymers using oxidative chemical vapor deposition (oCVD).^56–58^ This approach overcomes the limitations related to solution-based approaches, enabling us to profit from the huge potential offered by organic chemistry. Particularly, directly fused metalloporphyrin conjugated polymer thin films bearing different substituents (phenyl, mesityl, di(3,5-tertbutyl)phenyl and di(2,6-dodecyloxy)phenyl)^59^ and chelating different cations^25,58^ (Co(ii), Cu(ii), Ni(ii), Mg(ii), Zn(ii), Pd(ii), Pt(II), Ag(ii), Ru(ii), Ag(ii), and Fe(iii)) can be deposited over a wide variety of substates.

Herein, we report the preparation and engineering of efficient directly fused metalloporphyrin conjugated polymer electrocatalysts fulfilling the designing rules to operate at lower OER overpotentials. Among Earth abundant-transition metals, we have selected Ni(ii) as the chelated metal cation owing to its strong oxidizing potential, evidenced by the widely reported single-site electrocatalytic activity of Ni(ii)-based complexes, including porphyrins, toward the OER.^5,35–38,60–62^ Taking advantage of the versatility of the selected synthetic approach (oCVD), we demonstrate how the careful selection of the porphyrin substituent enables the optimization of the distance and interaction of water with the central metal cation, as well as facilitating O_2_ formation, to further enhance the electrocatalytic performances of the directly fused porphyrin conjugated polymer thin films. The influence of the substituent on the intermolecular and intramolecular coupling reaction and the electronic properties of the resulting thin films are evidenced by ultraviolet-visible-near infrared (UV/Vis/NIR) spectroscopy, laser desorption ionization high-resolution mass spectrometry (LDI-HRMS), X-ray photoelectron spectroscopy (XPS), and conductivity measurements, respectively. The thorough characterization of the thin films, complemented by density functional theory (DFT) calculations, enable the understanding of the influence of substitution on the electrocatalytic properties of the directly fused porphyrin conjugated polymer thin films.

## Results and discussion

### Synthesis and characterization of the fused porphyrin conjugated polymer thin films

**Scheme 1:**
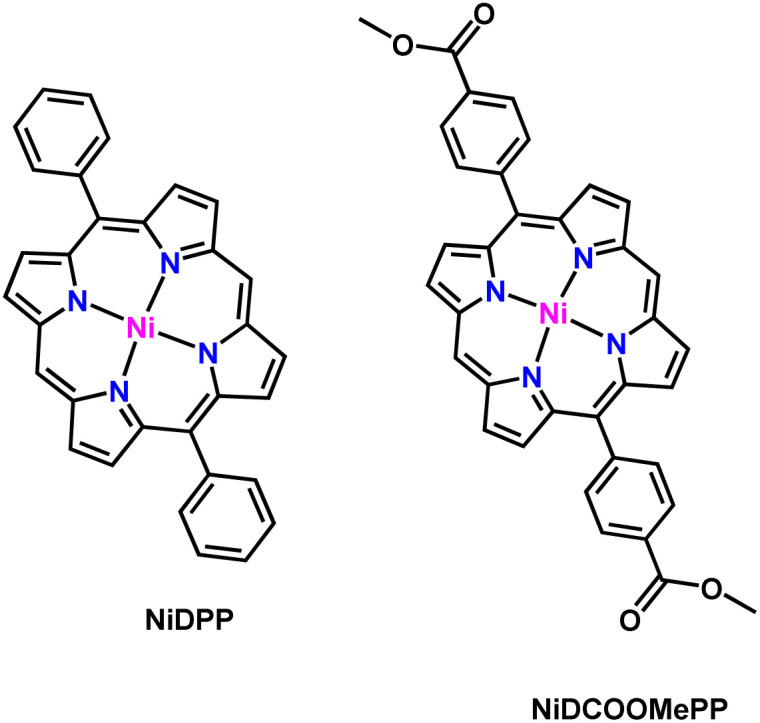
Molecular structures of the nickel(ii) porphyrins used in this work.

Fused porphyrin conjugated polymer thin films from Ni(ii) 5,15-(di-4-methoxycarbonylphenyl)porphyrin (pNiDCOOMePP) and Ni(ii) 5,15-diphenylporphyrin (pNiDPP) were prepared by oxidative chemical vapor deposition (oCVD)^56–58,63^ (see the Experimental section for details). Phenyl was selected as a *meso*-substituent due to its ability to undergo intramolecular dehydrogenative coupling with the β-position of the porphyrin macrocycle through its free *ortho*-position.^56^ Such extension of the π-electron conjugation of the fused porphyrin conjugated polymer was previously shown to yield a decrease of the overpotential required for the hydrogen evolution reaction (HER).^50^ 4-Methoxycarbonylphenyl, also possessing free *ortho*-positions and bearing the electron withdrawing ‘COOMe’ group, was also investigated to further increase the electrocatalytic properties of fused porphyrin conjugated polymers. The molecular structures of both investigated Ni(ii) porphyrins are presented in Scheme 1.

The oCVD reactions were performed in a custom-built reactor (Fig. S1†), using Fe(iii) chloride as the oxidant. Prior to the oCVD experiments, thermogravimetry analysis (TGA) was carried out to determine the optimal sublimation temperature of the porphyrin's monomers (Fig. S2†). All conditions for the preparation of the thin films reported in this work, such as the monomer and oxidant sublimation temperature, amounts consumed, reaction time and substrate temperature, are summarized in Table S1 of the ESI.† For the sake of comparison, thin films of the sublimed porphyrins (in the absence of the oxidant agent) were prepared under the same conditions and were denoted as sNiDCOOMePP and sNiDPP. Different substrates (microscope glass slides, silicon wafers, chips patterned with interdigitated electrodes and FTOs) were used toward further characterization of the deposited thin films.

**Fig. 1:**
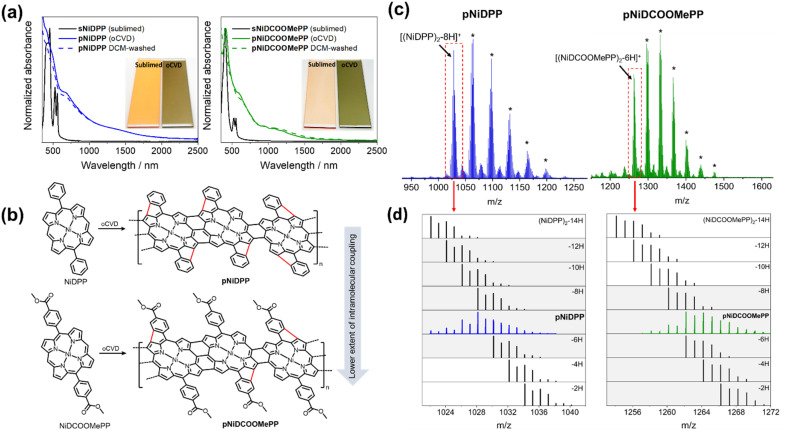
Synthesis of the fused porphyrin conjugated polymers. (a) UV/Vis/NIR absorption spectra of the as-deposited oCVD films for pNiDPP (blue solid line) and pNiDCOOMePP (green solid line) and after rinsing with dichloromethane (DCM) (dashed lines). The UV/Vis/NIR absorption spectra of the reference sublimed coatings (in the absence of an oxidant) are provided for comparison (black solid lines). Inset: digital pictures of the sublimed and oCVD thin films. (b) Representation of the oCVD reaction of both investigated 5,15-di-substituted Ni(ii) porphyrin monomers leading to fused porphyrin conjugated polymers, illustrating the lower extent of intramolecular coupling in pNiDCOOMePP as compared to pNiDPP, attributed to the electron withdrawing nature of the ‘COOMe’ group. Other side-reactions (*e.g.* chlorination) are not presented for clarity. (c) LDI-HRMS spectra in the dimeric region of the oCVD coatings pNiDPP (blue) and pNiDCOOMePP (green). The signals marked with the stars (*) correspond to chlorinated dimers [(NiDPP)_2_–(8 + *n*)H + *n*Cl]^+^ and [(NiDCOOMePP)_2_–(6 + *n*)H + *n*Cl]^+^. (d) Zoomed region evidencing the formation of dimeric species from the dehydrogenative C–C coupling from comparison with simulated isotopic patterns (black).

Both oCVD thin films, *i.e.*, pNiDCOOMePP and pNiDPP, exhibited an intense green color in comparison to orange-coloration of the just sublimed thin films, *i.e.*, sNiDCOOMePP and sNiDPP (Fig. 1a, inset), suggesting the successful oxidative dehydrogenative coupling of the porphyrins during the oCVD reaction, illustrated in Fig. 1b. The UV/Vis/NIR spectra of oCVD thin films, presented in Fig. 1a, display significantly broadened Soret and Q-bands as compared to the sharp absorption features of the sublimed thin films. One should note here that sNiDPP, formed from the sublimation of NiDPP, also exhibits a broadened Soret band attributed to the π–π stacking of the porphyrin units. Such a broadening is not observed for sNiDCOOMePP, which possesses bulkier substituents that hinder π–π stacking.

Additionally, the oCVD thin films show significant absorption in the visible and NIR regions indicating the formation of directly fused porphyrins. Consistent with a previous report, pNiDPP exhibits significantly broadened and red-shifted Soret and Q-bands along with a broad absorption feature between 1200 nm and 1600 nm, suggesting a high extent of the dehydrogenative coupling reaction, including intramolecular cyclisation and chlorination.

On the other hand, alongside a broadening of the Soret- and Q-bands, pNiDCOOMePP also displays more defined absorption features at 950 nm, 1077 nm, and 1184 nm that suggest the formation of oligomers/polymers with low intramolecular dehydrogenative C–C coupling, as presented in Fig. 1b. Indeed, fused porphyrin conjugated polymer thin films prepared from Ni(ii) porphyrins bearing aryl substituents with occupied *ortho*-positions, *e.g.*, mesityl, that prevent intramolecular cyclisation, also display such well-defined absorption bands between 800 nm and 1500 nm.^59^ In the present case, the low occurrence of intramolecular cyclisation in pNiDCOOMePP can be attributed to the electron withdrawing nature of the ‘COOMe’ group. It is worth noting here that the film thickness of both sublimed and oCVD coatings from NiDPP (*ca.* 200 nm, Table S2†) is almost four times higher than those from NiDCOOMePP (*ca.* 50 nm, Table S2†). It is assumed that the poor π-stacking ability of pNiDCOOMePP, arising from the structurally bulkier substituent, potentially leads to a lower sticking coefficient of the NiDCOOMePP monomer units that both adsorb at a lower rate and may desorb prior to their polymerisation during deposition process.

LDI-HRMS of both oCVD thin films revealed the formation of oligomers of Ni(ii) 5,15-diphenylporphyrin and Ni(ii) 5,15-(di-4-methoxycarbonylphenyl)porphyrin (Fig. 1c and S3†). Well-defined patterns corresponding to tetrameric species, alongside weak signals for pentameric species, are notably observed within the analytical limit of the instrument, *i.e.* 4000 *m/z*. Additionally, chlorination (a side reaction occurring when using chlorinated oxidants such as FeCl_3_ or CuCl_2_)^57^ is clearly observed in all the oCVD thin films from the peak distributions that shifted from *ca.* 35 *m/z* corresponding to the exchange of hydrogen atoms by chlorine. On the other hand, sublimed thin films sNiDCOOMePP and sNiDPP do not reveal the formation of any oligomeric/polymeric species (Fig. S4†). Importantly, as shown in Fig. 1c and d, the dimer region for pNiDPP is dominated by peaks related to M_2_–8H (*e.g.*, 1022.09 *m/z*), which necessarily implies the formation of intramolecular bonds. Peaks corresponding to M_2_–14H, associated with triply fused β–β/*meso*–*meso*/β–β dimers with 4 intramolecular cyclisation, are even observed (fully unsaturated dimers). Meanwhile, in the case of pNiDCOOMePP a lower extent of dehydrogenative coupling is suggested by the predominance of the peaks related to M_2_–6H (*e.g.*, 1262.183 *m/z*). This corroborates with the UV/Vis/NIR observation and points to a reduced intramolecular dehydrogenative coupling in pNiDCOOMePP as compared to pNiDPP, as a consequence of the electron withdrawing nature of the ‘COOMe’ group.

The morphology of the films prepared by oCVD was assessed by scanning electron microscopy (SEM) and transmission electron microscopy (TEM) analyses. The SEM images of the oCVD thin films (Fig. S5†) show a homogeneous coverage and relatively smooth and dense surface, in agreement with previous reports.^59,64,65^ The particles and islands disseminated across the surfaces are attributed to the inclusions of unreacted oxidant and oxidant by-products.^63^ TEM analysis (Fig. S6†) evidences a mixed amorphous and polycrystalline character for both polymers. The interplanar distance in pNiDCOOMePP (5.4 Å), corresponding to the 010 plane, is in agreement with previously calculated interplanar distances for Ni(ii) porphyrin conjugated polymers.^65^ The smaller value observed for pNiDPP (2.8 Å) arises from the planarization of the molecule due to the extended intramolecular cyclization reaction previously evidenced by LDI-HRMS. Indeed, former XRD analysis and DFT calculations showed that the pNiDPP thin film exhibits signals related to interplanar distances smaller than 3 Å.^65^

X-ray photoemission spectroscopy (XPS) measurement exhibits the atomic composition of N : Ni close to the theoretical ratio of 4 : 1 in both oCVD thin films (Table S3†). The minor deviation from the theoretical values is potentially due to the incorporation of Fe and Cl from the oxidant agent. The two different chlorine features observed in the Cl 1s XPS spectra correspond to the presence of unreacted FeCl_3_/FeCl_2_ at lower binding energies, and organic chlorine bonded to porphyrin at higher binding energies (Fig. S7 and S8†).^57,63^ It is worth mentioning that the analysis of the XPS spectra of the metal region (Ni 2p) in both the reference and oCVD thin films suggests no alterations of the metal cation (Fig. S7 and S8†). The binding energy of the main Ni 2p_3/2_ core level at *ca.* 855.4 eV and the weaker multiplets at higher binding energies (between 857.0 and 859 eV) are consistent with the values reported for other Ni(ii) porphyrins.^56,66,67^ The shake-up satellite observed at *ca.* 863.0 eV has also been previously reported for β-substituted Ni(ii) porphyrins.^56^ Interestingly, all the spectra in the N 1s region show a main peak at *ca.* 399.0 eV, characteristic of the pyrrolic nitrogen in Ni(ii) porphyrins.^25,56,66^ The absence of signals at 400.0 eV and 397.9 eV binding energies, characteristics of amino- and imine-pyrrole nitrogen environments of free-base porphyrins, confirms the retention of the metal cation at the porphyrin core.

Furthermore, the formation of a polymeric/oligomeric structure considerably influenced the valence band maxima (VBM) of the fused porphyrin conjugated polymer thin films, as the two oCVD thin films exhibit a shift of the VBM towards lower binding energies (Fig. S9†). Particularly, the VBM shifts from 2.97 eV in sNiDPP to 1.42 eV for pNiDPP, while the VBM only shifts from 1.97 eV in sNiDCOOMePP to 1.78 eV for pNiDCOOMePP (Fig. S10†). This is in agreement with our previous observation of increase in the energy of the Highest Occupied Molecular Orbital (HOMO) with the extension of π-electron delocalization resulting in the reduction of the band gap for multiply fused porphyrin conjugated polymer thin films. Importantly, the observation of a higher uplift of the VBM in pNiDPP in comparison to pNiDCOOMePP confirms a greater extent of dehydrogenative coupling in pNiDPP.

**Fig. 2:**
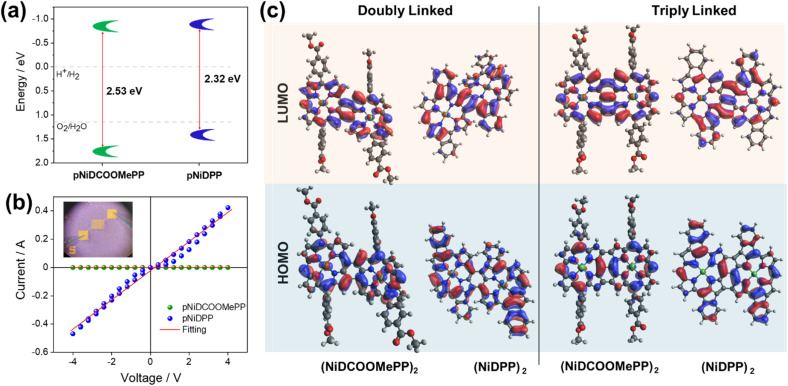
Electronic properties of the fused porphyrin conjugated polymers. (a) Schematic illustration of the band structures of the fused porphyrin thin films. (b) Lateral conductivity plot of pNiDCOOMePP and pNiDPP. (c) Optimized structures of doubly and triply fused NiDPP and NiDCOOMePP dimers showing the distribution of frontier molecular orbitals.

The energy band diagram of the fused porphyrin conjugated polymer thin films, constructed from the Tauc plot of the UV/Vis/NIR spectra (Fig. S11†) in combination with the VBM position calculated from the XPS analysis (Fig. S9†), is presented in Fig. 2a. Consistently, pNiDPP exhibits an optical band gap (*E*_g_ = 2.32 eV) smaller than pNiDCOOMePP (*E*_g_ = 2.53 eV), resulting from the more prominent, red-shifted absorption spectra. Both oCVD thin films possess suitable energy band positions to drive the electrocatalytic water splitting reactions. Interestingly, pNiDCOOMePP depicts a deeper VBM position, meaning a higher thermodynamic potential to drive the water oxidation, anticipating a superior activity as a heterogeneous OER catalyst.

Additionally, oCVD is known for the formation of conductive polymers by the extension of the conjugated system,^56–58^ a desired quality in catalyst materials for improved charge transport and transfer. The two oCVD thin films display an electrical conductivity several orders of magnitude higher than their respective sublimed thin films. However, lateral conductivity measurements on the oCVD thin films also point to a significantly lower conductivity for pNiDCOOMePP (4 × 10^−6^ S cm^−1^) in comparison to pNiDPP (3 × 10^−2^ S cm^−1^) (Fig. 2b). This is attributed to the bulkier nature of the substituent in pNiCOOMePP as compared to pNiDPP (hindering π–π stacking),^59^ but also to the lower degree of intramolecular cyclization induced by the 4-methoxycarbonylphenyl group, which results in a lower extent of electronic delocalization and further hinders π–π stacking.^59^ The higher conductivity of pNiDPP confirms a higher extension of the conjugated system, supported by the greater VBM shift, mainly due to the occurrence of intramolecular cyclization. However, such a high shift compromises the VBM position, getting closer to the water oxidation potential and, consequently, reducing the thermodynamic potential for the OER. In contrast, with a moderate shift, the VBM in pNiCOOMePP remains deep enough to provide a high thermodynamic potential for water oxidation.

To get further insights into the electronic properties of the oCVD thin films, DFT calculations (BP86-RIJCOSX-D3BJ/def2-TZVP) were performed with representative doubly and triply fused dimers (Fig. 2c) of NiDCOOMePP_2_ (*i.e.* (NiDCOOMePP)_2_ with no intramolecular cyclization and NiDPP (NiDPP)_2_ with intramolecular cyclisation). Unsurprisingly, structural optimization revealed out of plane aligned methoxycarbonylphenyl synthon in (NiDCOOMePP)_2_ and in-plane phenyl rings in (NiDPP)_2_ w.r.t the plane Ni(ii) porphyrin macrocycle. The influence of intramolecular cyclization is clearly seen on the distribution of Frontier Molecular Orbitals (FMOs) in both doubly and triply fused (NiDCOOMePP)_2_ and (NiDPP)_2_. While HOMO–LUMO surfaces are delocalized on the porphyrin units *via* C–C linkage in (NiDCOOMePP)_2_, the delocalization of FMOs is considerably extended on the phenyl rings in (NiDPP)_2_, in accordance with the increased conductivity in pNiDPP as compared to pNiDCOOMePP.

### Electrocatalytic activity towards the OER

**Fig. 3:**
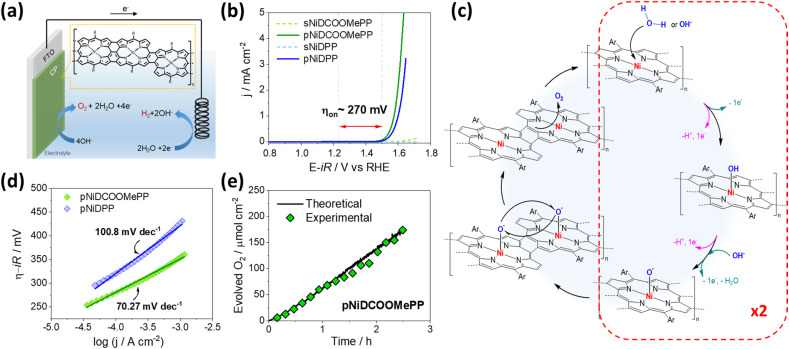
Electrocatalytic properties of the fused porphyrin conjugated polymers. (a) Representation of the OER electrode based on directly fused Ni–porphyrin conjugated polymers coated on FTO substrates. (b) Linear sweep voltammogram recorded at 100 mV s^−1^, in 1 M KOH, of the oCVD and sublimed films from Ni(ii) 5,15-(di-4-methoxycarbonylphenyl)porphyrin (NiDCOOMePP) and Ni(ii) 5,15-(di-phenyl)porphyrin (NiDPP). Potentials are *iR* drop-corrected (*R* = 20 Ω). (c) Representation of the radical oxo-coupling (ROC) pathway, in both acidic (pink) and alkaline conditions (green), proposed for the electrocatalytic OER activity of the oCVD thin films. (d) Tafel plots derived from linear sweep voltammetry of the oCVD thin films, indicating the calculated slope. (e) Evolved O_2_ amount determined by gas chromatography during the operation of pNiDCOOMePP (green diamonds), and theoretical amount derived from Faraday's law (black line).

Multiple Ni(ii) complexes, including porphyrins, have been investigated as homogeneous^5,61,62^ or heterogeneous^39,60^ electrocatalysts for water oxidation. However, Ni(ii) complexes of fused porphyrin conjugated polymers have not yet been investigated as catalysts due to synthetic challenges that were only recently overcame by the proposed oCVD approach.^25,56–59^ In this section, we assessed the electrocatalytic performance toward the OER for directly fused Ni(ii) porphyrin conjugated polymers coated on a FTO substrate, as presented in Fig. 3a. The linear and cyclic voltammogram of pNiDCOOMePP and pNiDPP in a 1 M KOH solution at pH 13.6, depicted in Fig. 3b and S12,† respectively, showed a significant electrocatalytic activity towards the OER for both the oCVD thin films, in contrast with the sublimed reference thin films. Indeed, the sublimed porphyrins (in the absence of the oxidant) were also measured as references, showing no significant OER activity. These observations confirm the significance of the dehydrogenative coupling reaction to enhance the electronic properties of Ni(ii) porphyrins,^56^ and therefore their performance as electrocatalysts.

It is worth noting that the activity of the thin films for water oxidation (2H_2_O → O_2_ + 4H^+^ + 4e^−^) was also evaluated in a 0.5 M Na_2_SO_4_ solution at pH 6.08 (Fig. S13†). However, the catalytic activity of both oCVD thin films is significantly lower than in 1 M KOH media. Indeed, previous reports on the OER using Ni(ii) porphyrins highlighted that they were more chemically stable in an alkaline electrolyte.^39,60^ In addition, the dissolution of the metal centre due to proton attack (porphyrin demetallation) is estimated to occur from a minimum pH of 2.63 for Ni porphyrins.^68^ Therefore, alkaline conditions were used for further characterization.

Interestingly, both films showed an onset overpotential to the OER potential (1.23 V *vs.* RHE) of *ca.* 270 m, comparable to recent reports of the electrocatalytic OER with cationic Ni(ii) porphyrin-based polymers coated on carbon nanotubes,^39^ and deformed Ni(ii) porphyrins immobilized on glassy carbon electrodes.^60^ Such a low overpotential suggests a possible dinuclear radical oxo-coupling (ROC) mechanism for the formation of O_2_, as presented in Fig. 3c, which allows lower overpotential values than the fundamental minimal required by the mononuclear water nucleophilic attack (WNA) pathway *i.e.*, 300 mV (Fig. S14 and S15†).^4,5^ These observations highlight the significant role of the direct fusion reaction in the formation of pre-organized electrocatalysts able to operate *via* a dinuclear mechanism, and in consequence at low overpotential, in contrast to the sublimed monomer units operating *via* the WNA at higher overpotentials.

From the LSV data, the Tafel plot was derived, as depicted in Fig. 3d. A Tafel slope of 70.3 mV dec^−1^ was obtained for pNiDCOOMePP, indicating the fastest kinetics and in consequence, higher catalytic performance than pNiDPP, deriving a Tafel slope of 100.8 mV dec^−1^. This points to an important contribution of the substituent to the electrocatalytic activity. Generally, the rate-limiting step in the ROC pathway is the interaction between the metal-oxo sites to form the O–O bond and further O_2_ release.^1^ Recently, Rao and co-workers discussed how the rate of water oxidation might be affected not only by the binding energy of the oxygen-containing intermediates, but also by the atomic arrangement of the active sites, which can facilitate oxo-coupling through geometric effects.^69^ As mentioned above, the withdrawing character of the ‘COOMe’ group reduces the occurrence of intramolecular cyclization in pNiDCOOMePP, allowing a greater flexibility and freedom for the Ni–O centres to move closer together and facilitate the oxygen radical coupling step to form O_2_*via* a ROC pathway. In contrast, pNiDPP is characterized by a strong extension of the intramolecular coupling reaction (Fig. 1c and d) resulting in a less adaptable structure due to its planar geometry. Importantly, a considerable spin density at the oxygen atom of the metal-oxo species is an essential condition for the formation of the O–O bond in the ROC mechanism.^4^ In this regard, the withdrawing character of the ‘COOMe’ group is expected to contribute towards the destabilization of the π-bond at the Ni–O species, increasing the spin density at the oxygen atom and thus, improving the reaction rate of the OER.

**Fig. 4:**
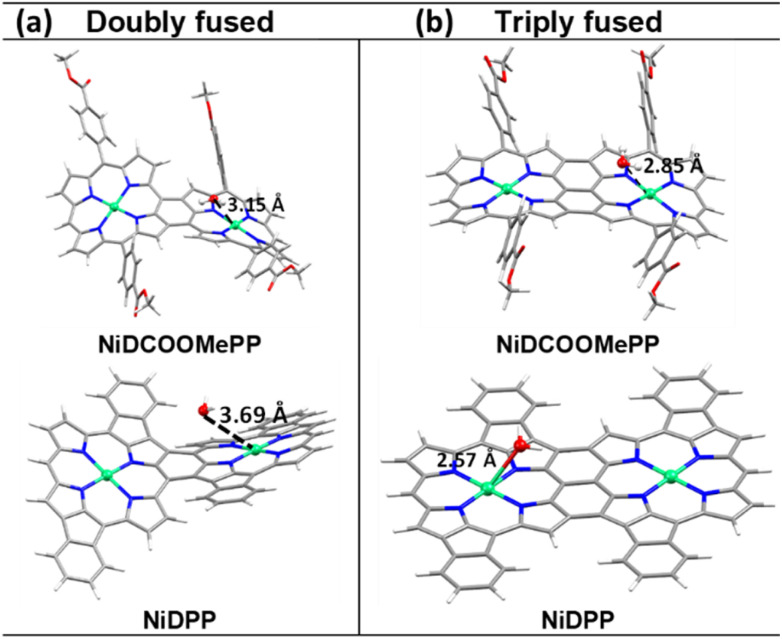
Water docking distance calculations: (a) docking results showing the interaction of optimized doubly and (b) triply fused (NiDCOOMePP)_2_ and (NiDPP)_2_ with the water molecule.

On the other hand, as depicted in Fig. 3c and S14,† the initial step of the ROC mechanism is the water molecule deprotonation and adsorption in the metal centre, (or OH^−^ electro-adsorption under alkaline conditions), both resulting in the formation of a *M–OH intermediate.^4,70^ Thus, it is of great importance in evaluating the ability of water to approach the active centre (Ni) and the ability of the product O_2_ to leave the Ni centres. Therefore, to decipher the role of the substituent in the electrocatalytic activity of fused Ni(ii) porphyrins, docking simulations between the water molecule and optimized dimeric structures were performed to predict their possible interaction and further activity towards OER. As shown in Fig. 4a, the docking studies show nicely positioned water molecules in close proximity to the catalytically active Ni(ii) centres of the optimized structures. The Ni⋯O_water_ distance is 3.15 Å in doubly fused (NiDCOOMePP)_2_, which increases to 3.69 Å in doubly fused (NiDPP)_2_. In contrast, triply fused (NiDPP)_2_ displays close interaction between Ni(ii)⋯O_water_ of 2.57 Å, whereas in (NiDCOOMePP)_2_ and similarly to its doubly fused counterpart, the water molecule remains at an optimal distance to the Ni(ii) centre (Ni⋯O_water_ = 2.86 Å). Notably, the effective Ni(ii)⋯O_water_ interaction without forming a strong bond is deemed crucial for the release of the oxygen molecule after the water oxidation reaction. Thus, from the docking analysis, pNiDCOOMePP should be more appropriately spaced from the water molecule compared to pNiDPP, which in combination with the withdrawing role of the ‘COOMe’ group contributing to enhance the radical character of the oxygen atom anticipates a faster release of O_2_ from the pNiDCOOMePP catalyst. On the other hand, in the case of pNiDPP, the formation of a strong bond between Ni(ii) and O_water_ most likely leads to the formation of NiO_*x*_ species or slow release of O_2_. It is worth noting that the acquisition of consistent evidence on the electronic state of the nickel centre under operating conditions is quite challenging;^71^ therefore, the elucidation of the precise geometry and electronic structure of the Ni–O species is not straightforward.

Interestingly, although it has both a lower thickness (*ca.* 47 nm *vs.* 207 nm) and a lower conductivity (*ca.* 4 × 10^−6^ S cm^−1^*vs.* 3 × 10^−2^ S cm^−1^), the electrocatalytic properties of pNiDCOOMePP outperform the electrocatalytic properties of pNiDPP, which can be attributed to the combination of three factors: (i) the lower degree of conjugation in pNiDCOOMePP, allowing the retention of the VBM position deep enough to provide a high thermodynamic water oxidation potential; (ii) the facilitation of the interaction between the Ni–O sites and enhancement of the spin density at the oxygen atom to form the O–O bond *via* the ROC pathway; and (iii) the optimal water interaction with the central metal cation resulting from the advantageous molecular conformation of the pNiDCOOMePP catalyst due to the presence of the ‘COOMe’ substituents.

The stability of the two oCVD thin films towards the OER was evaluated by both chronoamperometric and potentiometric measurements (Fig. S16a and b†). Notably, a gradual increase in the current density is observed during the chronoamperometry test, possibly associated with a transient behaviour, *e.g.*, dynamic surface rearrangement under applied potential conditions. Interestingly, an increase of the current density under similar conditions was previously reported for Ni(ii) porphyrin-modified glassy carbon electrodes, which was associated with possibly improved both active site accessibility and hydrophilic character of the electrocatalyst at applied potential for a longer time.^60^

**Fig. 5:**
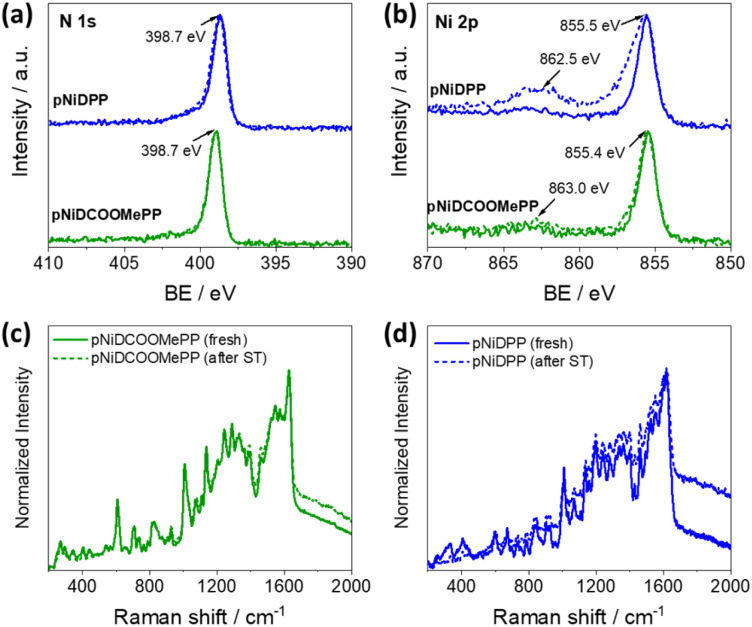
Post-operational characterisation. (a and b) XPS spectra showing the N 1s and Ni 2p core level region of the catalyst, respectively, before (straight line) and after (dashed line) the stability test (ST). (c and d) Raman spectra of the pNiDCOOMePP and pNiDPP catalysts, respectively, before (straight line) and after (dashed line) the stability test.

XPS, Raman spectroscopy, and LDI-HRMS analyses were performed to verify the post-operational structural and chemical stability of the fused Ni(ii) porphyrin conjugated polymer thin films. Notably, as depicted in Fig. 5a and b, no significant alteration on the N 1s and Ni 2p core level regions in pNiDCOOMePP was observed, pointing to the retention of the Ni(ii) metal cation inside the porphyrin macrocycle after electrochemical operation. Indeed, analysis on the N 1s region (Fig. 5a) showed the characteristic signal at 398.7 eV, corresponding to pyrrolic nitrogen in Ni(ii) porphyrins, pointing to the absence of demetallated porphyrin units in pNiDCOOMePP. Moreover, the binding energy of the main Ni 2p_3/2_ core level, centred at *ca.* 855.4 eV (Fig. 5b), is characteristic of Ni(ii) porphyrins.^56,66,67^ The atomic composition of N : Ni also remains close to the theoretical ratio, 4 : 1 (Table S4†). On the other hand, although the N 1s core level spectrum of pNiDPP remains unaltered after the stability test, the Ni 2p_3/2_ core level envelope is broadened to higher binding energies and the intensity of its shake-up satellite peak significantly increased, pointing to an increase of the oxidation state of nickel. As anticipated by the docking experiments, the lower water docking distance to the Ni centre in the pNiDPP catalyst possibly leads to strongly bonded intermediates (*Ni–OH). In contrast, the optimal water docking distance predicted for pNiDCOOMePP facilitates the efficient release of oxygen atoms in the form of O_2_.

The Raman spectra of both films before and after the stability test largely overlap, and no new phases are observed, as shown in Fig. 5c and d. Moreover, comparative LDI-HRMS analysis of the electrodes confirmed the presence of porphyrin oligomers (Fig. S17a and b†), indicating the retention of the Ni(ii) porphyrin conjugated polymer structures on the electrode surface after operation. Although the post-operational characterization pointed to the preservation of the catalyst structure, additional *in situ* characterization is required to provide specific information on the possible formation of surface transition states and/or intermediates during the reaction process.

Finally, the amount of evolved oxygen at the pNiDCOOMePP surface was determined by gas chromatography. An Agilent gas chromatograph was coupled to the sealed electrochemical cell, provided with a constant flow of argon as the carrier gas. The oxygen evolution rate was evaluated at an overpotential of 400 mV, *i.e.*, 1.63 V *vs.* RHE of constant applied potential, while the driven current was recorded (Fig. S18a†) and further used to calculate the expected O_2_ amount from Faraday's law (see the Experimental section for details). As depicted in Fig. 3e and S18b,† O_2_ is produced with a faradaic efficiency close to 100% after 2.5 h of operation, with a maximum evolution rate of 69.6 μmol cm^−2^ h^−1^.

oCVD allows the preparation of pre-organized polymer catalysts with superior performance to their monomeric counterparts, due to the facilitation of a water oxidation mechanism operating at lower overpotential. Although the electrocatalytic current density obtained is moderated (below 10 mA cm^−1^) with respect to other Ni(ii) porphyrin-based systems,^39^ they are coherent with the smaller active surface area of the thin films deposited on FTO substrates in comparison with three-dimensional porous scaffolds (*i.e.* carbon nanotube supported porphyrins).^39^ Interestingly, the current densities achieved from both pNiDMeCOOPP and pNiDPP conjugated polymers are two orders of magnitude higher than that of Ni(ii) porphyrin drop-casted on glassy carbon electrodes (*ca.* 75 μA cm^−2^ at 1.7 V *vs.* RHE).^60^ Noteworthily, current density values reported in this work are in the same range as those observed for Co(ii) porphyrins coated on FTO substrates (*ca.* 3 mA cm^−2^ at 2.04 V *vs.* RHE).^48^ According to the present findings on the catalytic activity of Ni(ii) porphyrin conjugated polymers on FTO, future work can be therefore directed towards the optimization of the oCVD conditions to improve the porosity and subsequent catalytic robustness of porphyrin conjugated polymers. Interestingly, a previous study demonstrated that higher reaction temperatures can lead to a large decrease of the film density and to the formation of a mesoporous morphology of the fused porphyrin thin film on a planar substrate.^65^

Furthermore, the present results pave the way to the development and integration of fused porphyrin-based conjugated polymers as heterogeneous catalysts for sustainable fuel production. Particularly, the selection of a porphyrin pattern merging both previous^50–52^ and present findings, *i.e.*, the significance of the intramolecular cyclisation reaction for enhanced charge transfer, and the optimization of the band position and of the interaction between the reactant and the catalytic centre, should enable the further enhancement of the catalytic performance of polymer catalysts based on fused porphyrins.

## Conclusions

Fused Ni(ii) porphyrin conjugated polymer thin films have been successfully implemented as heterogeneous catalysts for OER. The porphyrin conjugated polymer thin films were demonstrated to be more kinetically and thermodynamically active than the sublimed (monomer) porphyrin thin films owing to the formation of long range conjugated structures enabling a dinuclear operational mechanism at low overpotential. The electron withdrawing character of the ‘COOMe’ group was demonstrated to be responsible for a lower extent of dehydrogenative intramolecular coupling in oligomer/polymer chains, leading to a lower degree of conjugation. As a result, the VBM can be retained at deep values, driving a higher thermodynamic oxidation potential. The molecular conformation of the pNiDCOOMePP polymer allows greater flexibility and freedom for the Ni–O centres to facilitate the oxygen radical coupling step, in contrast to the less adaptable structure of pNiDPP due to its planar geometry. Additionally, the withdrawing role of the ‘COOMe’ group contributes to increasing the radical character the oxygen atom, improving the reaction rate of OER. Finally, docking studies show a well-positioned water molecule at an optimum distance w.r.t Ni(ii) centres in pNiCOOMePP compared to pNiDPP for effective water oxidation catalysis. This work opens the path to the molecular design of efficient polymer catalysts and to further investigations on the operational mechanism for heterogeneous water oxidation catalysis using fused Ni(ii) porphyrin thin films.

## Experimental

### Thin film preparation by oxidative chemical vapor deposition (oCVD)

The oCVD reaction was performed in a custom-built oCVD reactor described in detail elsewhere.^56,57,63^ 5,15-Diphenyl Ni(ii)porphyrin and 5,15-(di-4-methoxycarbonylphenyl) Ni(ii)porphyrin were obtained from PorphyChem (98%) and were used without further purification. Based on previous reports,^56,57^ iron(iii) chloride (97%, Sigma Aldrich) was chosen as the oxidant. Table S1† summarizes the deposition conditions used for each porphyrin investigated. The temperature used to sublime the oxidant was 170 °C in all cases. Glass microscope slides (Menzel-Gläser Superfrost®), silicon wafers (Siegert Wafer®), interdigitated chips (OFET Gen4, Fraunhofer) and fluorine-doped tin oxide (FTO) coated glass were used as substrates for further characterization. Prior to deposition, all the substrates were cleaned with absolute ethanol (99.98%, VWR Chemicals®) and dried with nitrogen gas. The substrate holder was kept at 150 °C for all the depositions. The pressure inside the oCVD reactor was kept at 10^−3^ mbar, under an argon (99.999%, Air Liquide) atmosphere. The deposition time was set to 30 minutes for all experiments. Additionally, reference sublimed porphyrin monomer films (in the absence of the oxidant) were obtained under the same conditions.

### Thin film characterization

The ultraviolet-visible-near infrared (UV/Vis/NIR) spectra of the sublimed and oCVD films deposited on glass slides were recorded with in a PerkinElmer Lambda 1050 spectrometer, in the transmission (*T*) mode, in the 300–2500 nm wavelength interval. The absorbance (*A*) was calculated as: *A* = −log(*T*). From the absorbance spectra, the direct optical band gap of the fused-metalloporphyrins was estimated through the Tauc plot as: (*αhν*)^1/*n*^ = *A*(*hν* − *E*_g_), where *α* is the absorbance coefficient, *n* = 1/2 for direct transitions, *h* is the Planck's constant and *ν* the wavelength number. The absorbance coefficient was calculated as: *α* = ln(10)*A*/*l*, where *l* is the film's thickness. The thin film thicknesses were measured using a KLA-Tencor P-17 Stylus Profiler. Additionally, the thin films were rinsed with dichloromethane (HPLC grade >99.8%, SupraSolv®) for comparison with the as-deposited films.

Scanning electron microscopy (SEM) images were recorded using a FEI Quanta 200F instrument. Transmission electron microscopy (TEM) analyses were performed with a JEOL JEM-F200 cold FEG microscope operating at an acceleration voltage of 200 kV. Energy dispersive spectroscopy (EDS) was carried out in STEM mode. For preparing the samples, the as-prepared films were scratched, and the material collected was dispersed in ethanol. A Cu mesh covered with a holey carbon film (AGS147-3H, Agar Scientific Ltd) was immersed in the dispersion and let dry until full evaporation of the solvent. X-ray photoelectron spectroscopy (XPS) measurements were performed with a Kratos Axis Ultra DLD instrument using a monochromatic Al K_α_ X-ray source of energy 1486.6 eV, at 105 W power. The analyses were performed under 5 × 10^−9^ mbar vacuum pressure. Charge calibration was accomplished by fixing the binding energy of carbon (C 1s) to 285.0 eV. Raman spectra were recorded at room temperature with an inVia Raman Microscope (RENISHAW), using 633 nm laser excitation. All the oCVD samples (as prepared and after electrochemical characterization) were stored under vacuum conditions until their subsequent analysis.

Laser desorption/ionization high-resolution mass spectrometry (LDI-HRMS) measurements were performed using an AP-MALDI UHR ion source (MassTech, Inc.) coupled to an LTQ/Orbitrap Elite (Thermo Scientific). In-source fragmentation (*E* = 70 V) was used to prevent the formation of clusters. The measurements were performed on Si wafer substrates and FTO-coated glass coated either with the sublimed porphyrin monomer and oCVD film, which were directly placed on the sample holder. LDI-HRMS measurements and analysis of metalloporphyrin coatings have been described in detail elsewhere.^57,58,63^

### Electrochemical characterization

Linear sweep voltammetry (LSV), cyclic voltammetry (CV) and chronoamperometry measurements were performed with an Autolab PGSTAT302 potentiostat/galvanostat, in a three-electrode configuration cell. The cell consisted of a Pt wire as the counter electrode, an Ag/AgCl (3 M KCl) electrode as the reference electrode, and the porphyrin coating on fluorine-doped tin oxide (FTO) coated glass as the working electrode. A 1 M potassium hydroxide (Sigma Aldrich) solution at pH 13.6 was used as the alkaline electrolyte. Alternatively, a 0.5 M sodium sulphate (Sigma Aldrich) solution at pH 6.08 was used as the electrolyte for measurements under acidic conditions. All the potentials were referenced to the Reversible Hydrogen Electrode (RHE) using the Nernst equation: *V*_RHE_ = *V*_Ag/AgCl_ + *V*^0^_Ag/AgCl _+ 0.0591 × pH.

The oxygen evolution at the surface of the oCVD thin films was determined by gas chromatography measurements using a sealed cell coupled to an Agilent Micro-GC gas chromatograph, during a chronoamperometric measurement at 1.63 V *vs.* RHE. The faradaic efficiency (FE) was estimated through the relationship: FE (%) = O_2_(exp)/O_2_(theo), where O_2_(exp) is the amount of evolved O_2_ in mol, monitored every 5 min, and O_2_(theo) is the theoretical O_2_ evolution calculated with Faraday's law: *n* (mol cm^−2^) = *j*_O_2__*t*/*nF*, where *j*_O_2__ is the current density recorded by chronoamperometry measurement, *t* is the time in seconds, *n* is the number of electrons transferred in the reaction and *F* is the Faraday constant, 96 485.33 C mol^−1^.

### Density functional theory calculations

All the structures were optimized using Orca version 5.0.1.^72–74^ The DFT calculations were performed using the BP86 (ref. 75 and 76) functional with the Karlsruhe valence triple-zeta basis set “def2-TZVP”^77–79^ and Weigend's auxiliary basis set.^80^ Dispersion effects were considered by Grimme approximation ‘D3’.^81,82^ To simply speed up the iteration, RIJCOSX approximation is included.^83,84^ The optimized geometries were confirmed attaining local minima by confirming the absence of negative frequencies after numerical frequency analysis. Docking experiments were performed using Hex 8.0 docking software. In all the DFT calculations, the Ni(ii) centre was considered in a low spin state.

## Author contributions

D. B.: synthesis, investigation, formal analysis, writing – original draft, and writing – review and editing; D. C.-M.: investigation, formal analysis, writing – review and editing; N. B: conceptualization, methodology, formal analysis, funding acquisition, and writing – review and editing.

## Conflicts of interest

There are no conflicts to declare.

## Supplementary Material

TA-011-D2TA07748E-s001
