# Supplementary material for: Conjugated porphyrin polymer films with nickel single sites for the electrocatalytic oxygen evolution reaction
Source: J Mater Chem A Mater. 2023 Jan 26;11(10):5188–98. doi: 10.1039/d2ta07748e (PMC9990145; doi:10.1039/d2ta07748e)
Supplement: TA-011-D2TA07748E-s001 [file TA-011-D2TA07748E-s001.pdf]

Electronic Supplementary Material (ESI) for Journal of Materials Chemistry A.
This journal is © The Royal Society of Chemistry 2023

Supporting Information

Conjugated Porphyrin Polymer Films with Nickel Single-Sites for Electrocatalytic Oxygen Evolution Reaction

Deepak Bansal,* Drialys Cardenas-Morcoso, Nicolas Boscher*

Materials Research and Technology Department, Luxembourg Institute of Science and Technology, 28 Avenue des Hautes-Fourneaux, Esch-Sur-Alzette, Luxembourg

**Email:* deepak.bansal@list.lu, nicolas.boscher@list.lu

Content	Page no.
**Fig. S1**. Schematic representation of the custom built oCVD reactor…………………………….	2
**Fig. S2**. Thermogravimetric analysis of the porphyrin's monomers………………………………	2
**Fig. S3**. LDI-HRMS spectra of **pNiDCOOMePP** and **pNiDPP**…………………………………	4
**Fig. S4**. Comparative LDI-HRMS spectra of oCVD films with the reference sublimed films…...	5
**Fig. S5.** SEM images of **pNiDCOMePP (a)** and **pNiDPP (b)** films……………………………..	5
**Fig. S6.** TEM images of **pNiDMeCOOPP ((a) & (b))** and **pNiDPP ((c) and (d))** film………..	6
**Fig. S7**. XPS spectra of oCVD **pNiDPP** coating…………………………………………........	7
**Fig. S8**. XPS spectra of oCVD **pNiDCOMePP** coating…………………………………….........	8
**Fig. S9**. Valence band minimum energy (VBM) determination of the reference and oCVD coatings……………………………………………………………………………………….	8
**Fig. S10**. Representation of the VBM (HOMO) shift from sublimed to oCVD films…………..	9
**Fig. S11**. Tauc's plots obtained from the UV-Vis-NIR absorbance of the oCVD coatings for energy band gap value estimation……………………………………………………………	9
**Fig. S12**. Cyclic voltammetry of the oCVD and sublimed films in 1M KOH……………………	10
**Fig. S13**. Cyclic voltammetry of the oCVD and sublimed films in 0.5M Na_2SO_4………………..	10
**Fig. S14**. Representation of the water nucleophilic attack pathway………………………………	11
**Fig. S15.** Energy barrier diagram, considering optimal operation of a catalyst operating via a WNA mechanism………………………………………………………………………..	11
**Fig. S16**. Chronoamperometry measurement on the oCVD film…………………………………	12
**Fig. S17.** Comparative LDI-HRMS spectra of **pNiDCOOMePP (a)** and **pNiDPP (b)** catalysts before (fresh) and after stability test.	13
**Fig. S18**. Chronoamperometry and gas evolution measurements on the **pNiDCOOMePP** film...	13
**Table S1**. Experimental details for both porphyrins investigated and the oxidant………………	3
**Table S2**. Thickness of the sublimed and oCVD coatings from NiDPP and NiDCOOMePP……	3
**Table S3**. Relative elemental composition from XPS analysis of the reference and oCVD coatings……………………………………………………………………………………….	7
**Table S4**. Relative elemental composition from XPS analysis of the oCVD thin films before and after chronoamperometry………………………………………………………………….	12
**Table S5-S8**. Coordinates for triply and doubly fused NiDCOOMePP and NiDPP dimer………	14-31

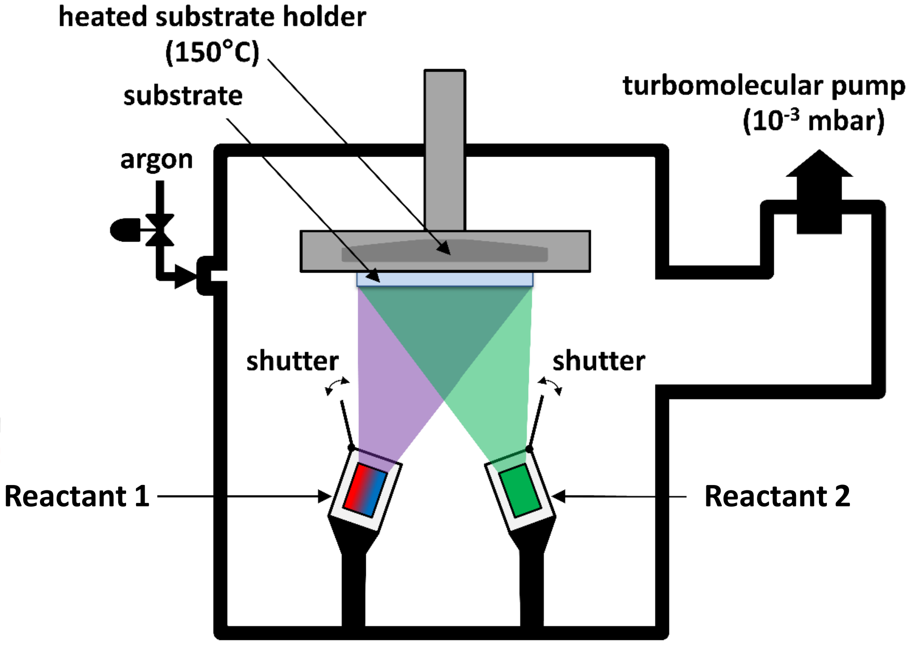

Fig. S1. Schematic representation of the custom built oCVD reactor used for the experiments. The metalloporphyrins (reactant 1) are sublimed under vacuum towards the substrate simultaneously to the oxidant ($FeCl_3$, reactant 2).

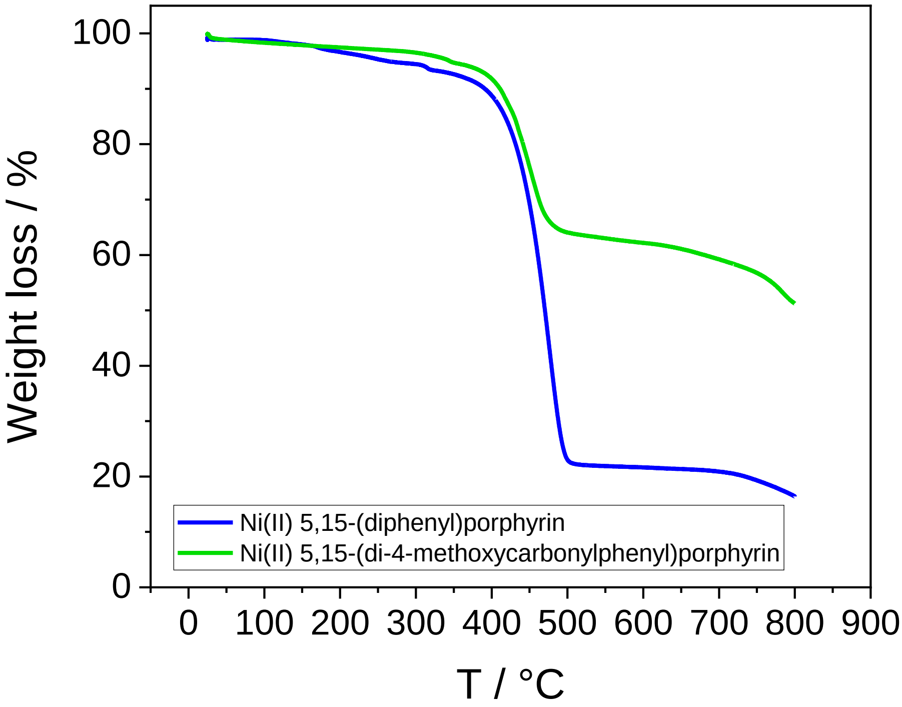

Fig. S2. Thermogravimetric analysis of the porphyrin's monomers.

Table S1. Chemical formula, molecular weight, sublimation temperature and sublimed amount for both porphyrins investigated and the oxidant. Substrate temperature was 150°C and working pressure was 10^{-3} mbar.

**NiDPP** – Nickel(II) 5,15-(diphenyl)porphyrin	
Chemical Formula	$C_{32}H_{20}N_4Ni$
Molecular Weight	519.23 g·mol^{-1}
Sublimed Temperature	240°C
Sublimed Amount	8.8 mg
**NiDCOOMePP** – Nickel(II) 5,15-(di-4-methoxycarbonylphenyl)porphyrin	
Chemical Formula	$C_{36}H_{24}N_4NiO_4$
Molecular Weight	635.305 g·mol^{-1}
Sublimed Temperature	290
Sublimed Amount	9.5 mg
**$FeCl_3$** – Iron(III) chloride	
Chemical Formula	Cl_3Fe
Molecular Weight	162.20 g·mol^{-1}
Sublimed Temperature	155°C
Sublimed Amount	133.8 mg for NiDPP<br>117.3 mg for NiDCOOMePP

Table S2. Thickness of the sublimed and oCVD coatings from NiDPP and NiDCOOMePP.

**Porphyrin**	Thickness (nm)
**sNiDPP**	235 nm
**pNiDPP**	204 nm
**sNiDCOOMePP**	57 nm
**pNiDCOOMePP**	47 nm

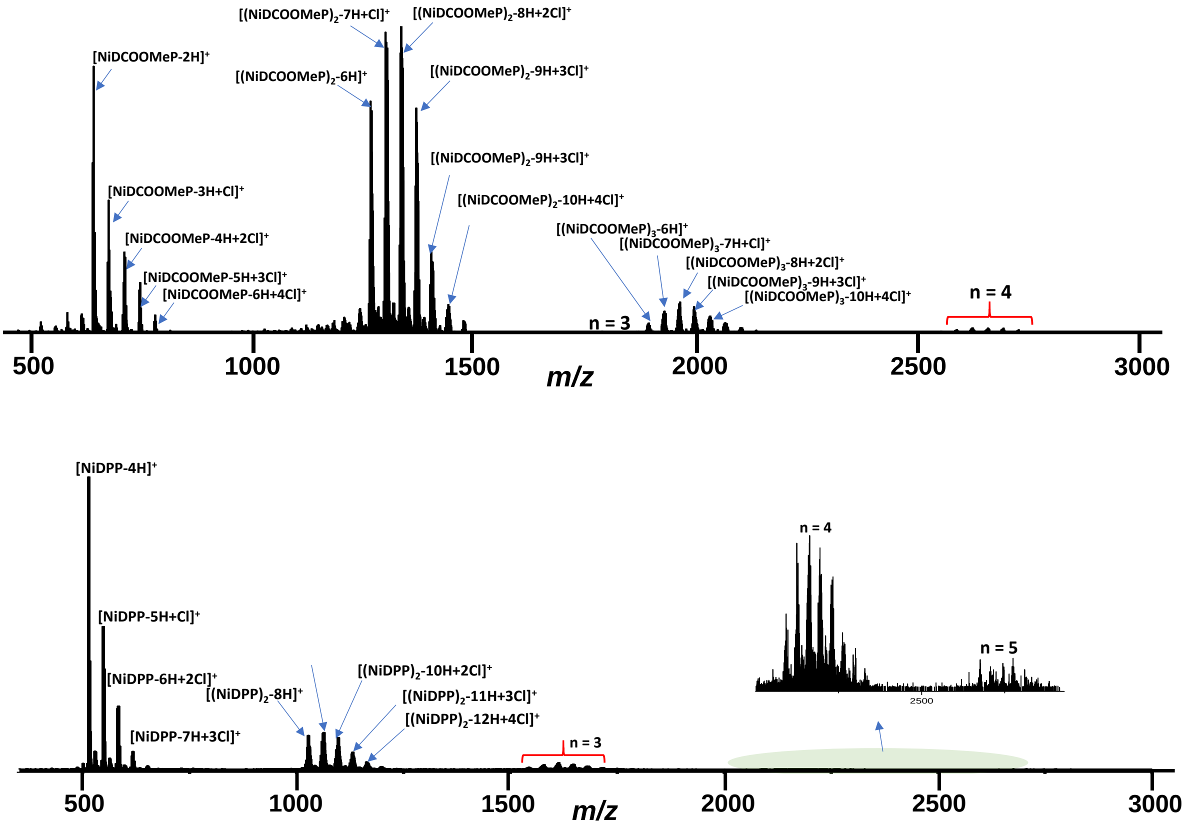

Fig. S3. LDI-HRMS spectra of **pNiDCOOMePP** (top) and **pNiDPP** (bottom) showing the presence of different oligomers from the C-C coupling reaction, along with chlorination side-reaction.

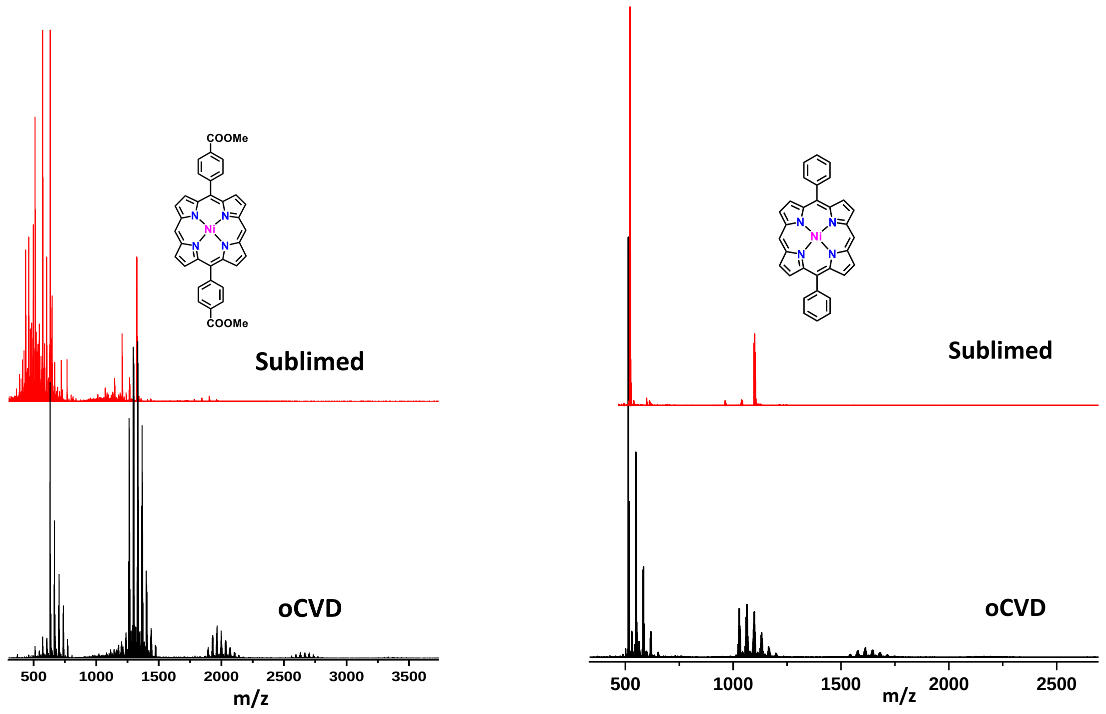

Fig. S4. Comparative LDI-HRMS spectra of oCVD thin films with the reference sublimed thin films prepared from NiDCOOMePP (left) and NiDPP (right) showing the absence of oligomeric features in sublimed materials in absence of $FeCl_3$.

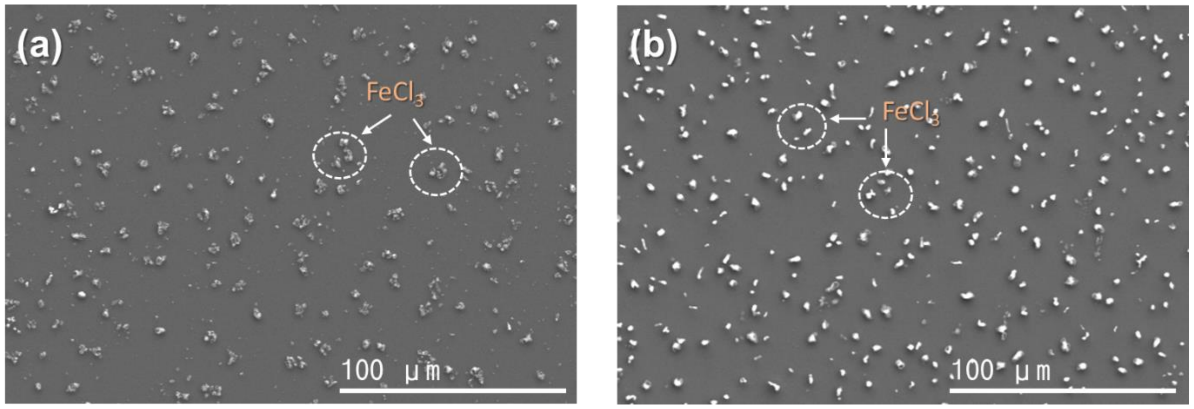

Fig. S5. SEM images of **pNiDCOMePP (a)** and **pNiDPP (b)** films.

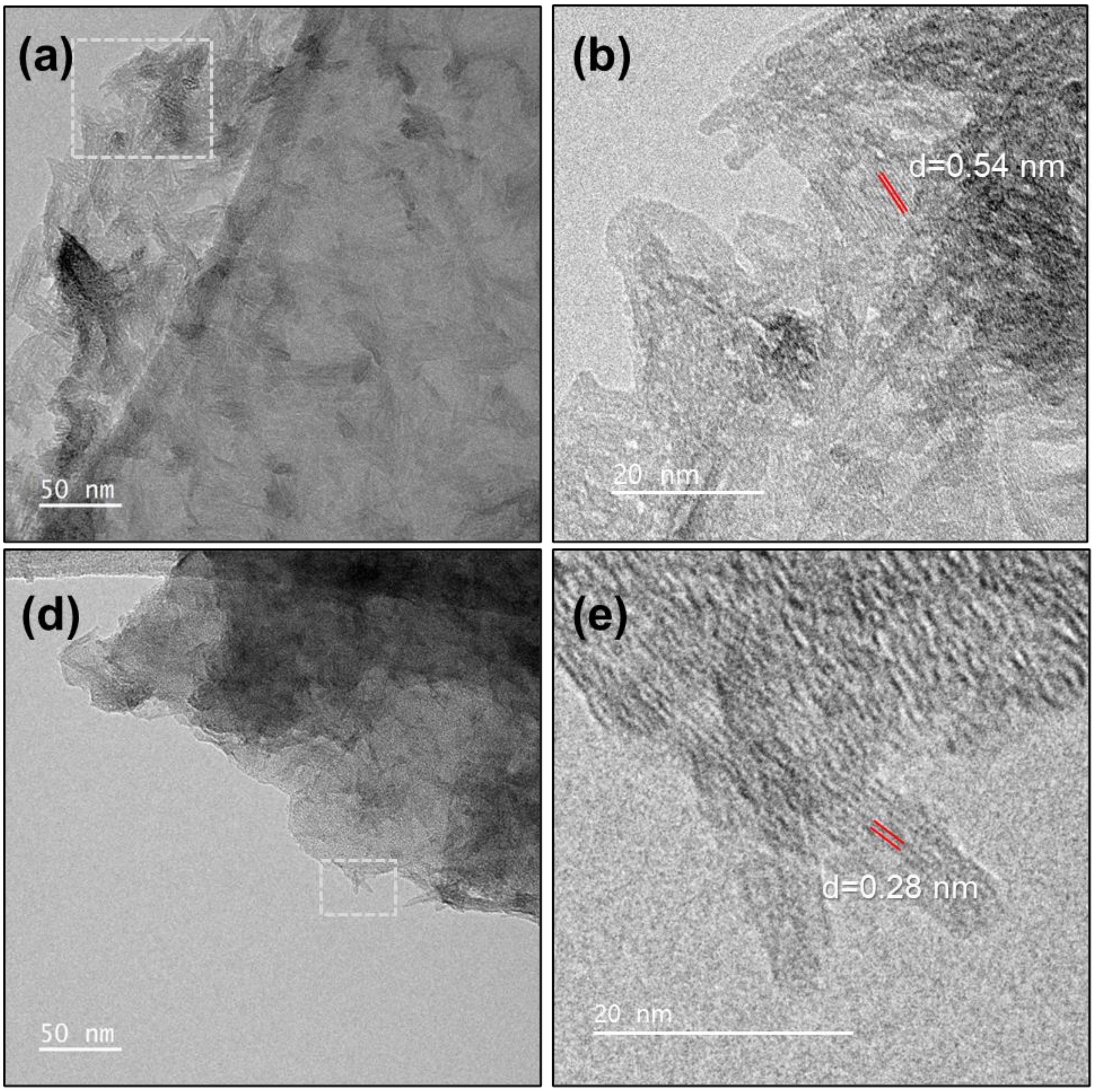

Fig. S6. TEM images of **pNiDMeCOOPP ((a) & (b**)) and **pNiDPP** (**(c) and (d)**) film.

Table S3. Relative elemental composition from XPS analysis of the reference (sublimed) and oCVD coatings.

Sample	C 1s at.%	N 1s at.%	Ni 2p at.%	O 1s at.%	Cl 2p at.%	Fe 3p at.%
**sNiDCOOMePP**	75.5	6.8	1.7	11.1	-	-
**pNiDCOOMePP**	74.7	4.9	1.1	13.6	3.5	2.3
**sNiDPP**	87.6	10.1	2.2	0.2	-	-
**pNiDPP**	77.4	6.4	1.5	9.0	3.3	2.5

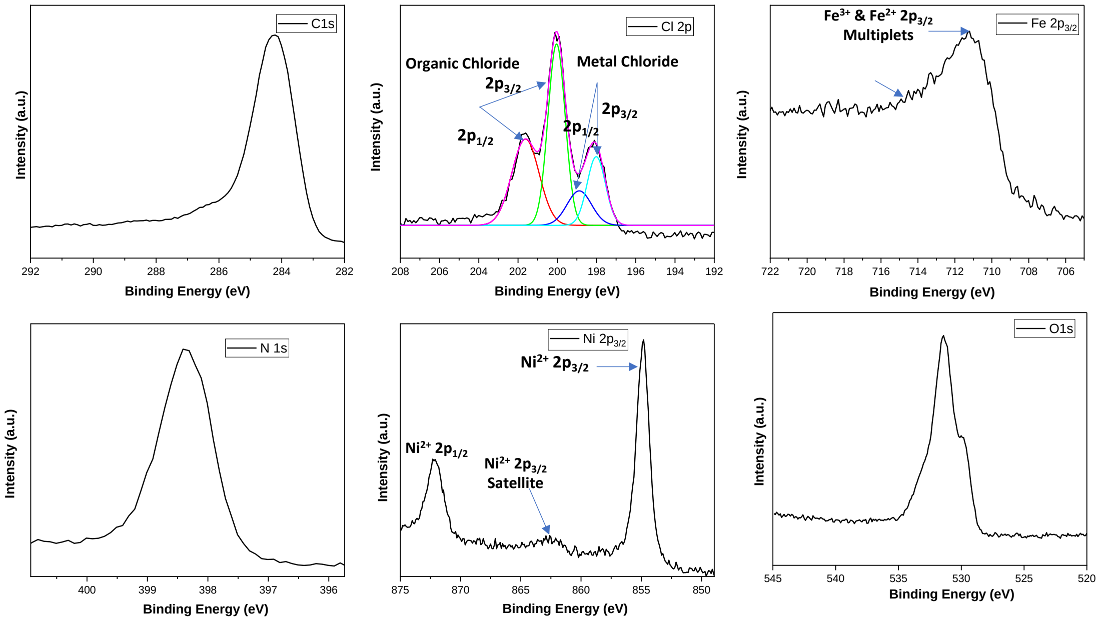

pNiDPP

Fig. S7. XPS spectra of the Ni $2p_{3/2}$, N 1s, C 1s, Fe $2p_{3/2}$, Cl 2p and O1s core levels of the oCVD **pNiDPP** coating.

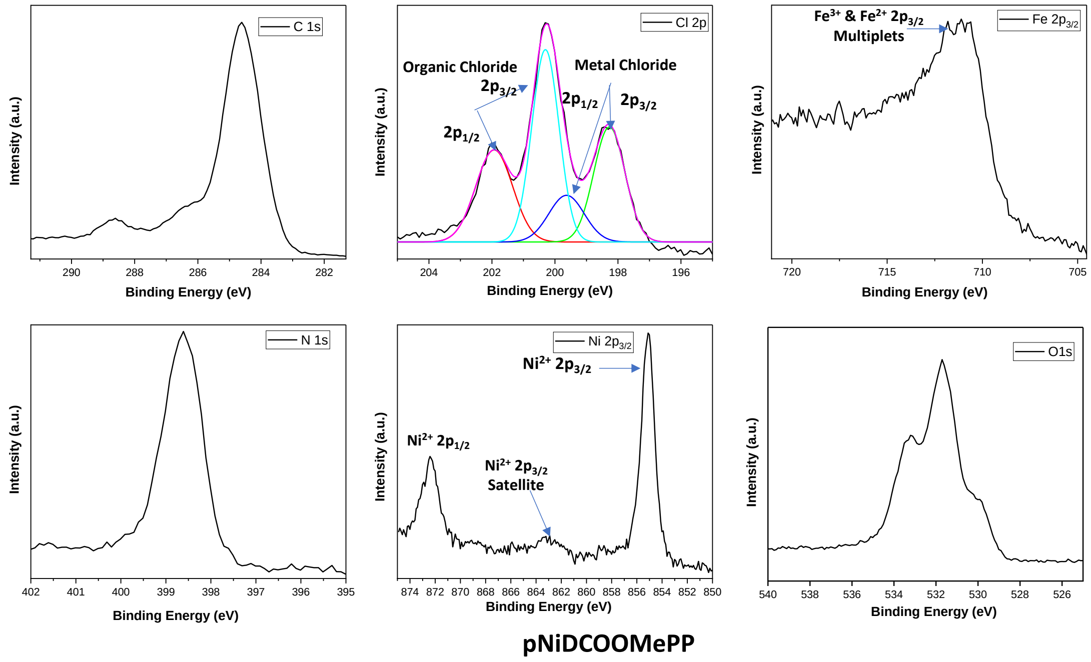

Fig. S8. XPS spectra of the Ni $2p_{3/2}$, N 1s, C 1s, Fe $2p_{3/2}$, Cl 2p and O1s core levels of the oCVD **pNiDCOOMePP** coating.

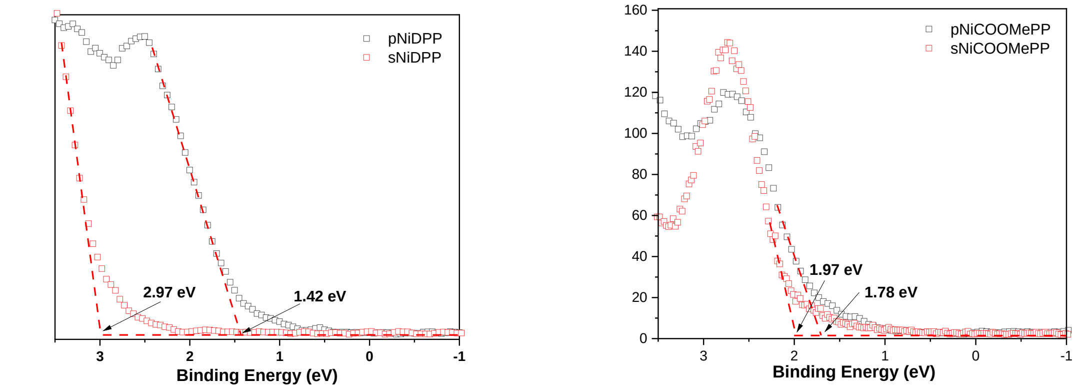

Fig. S9. Valence band minimum energy (VBM) determination in the valence band region of the XPS spectra of the reference (sublimed) (red squares) and oCVD (black squares) coatings.

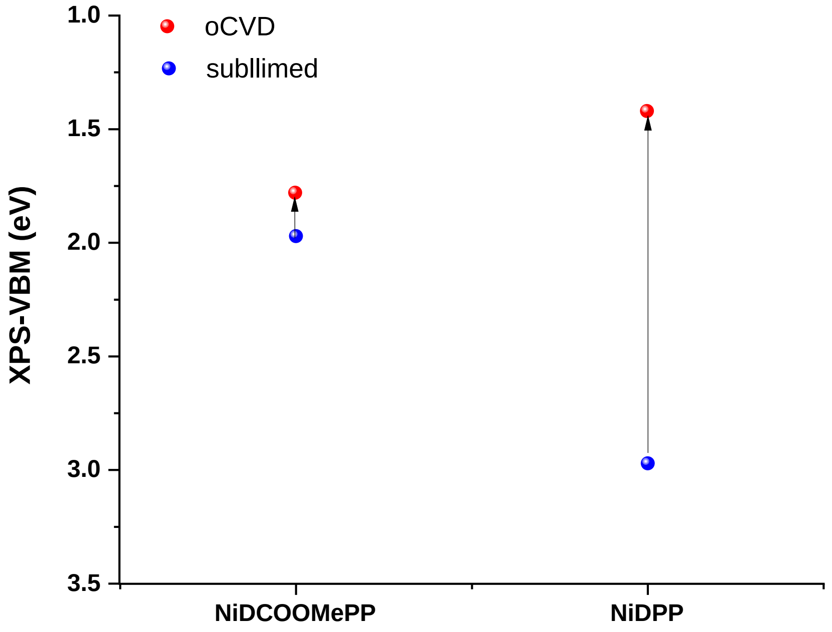

Fig. S10. Representation of the VBM (HOMO) shift from sublimed to oCVD films.

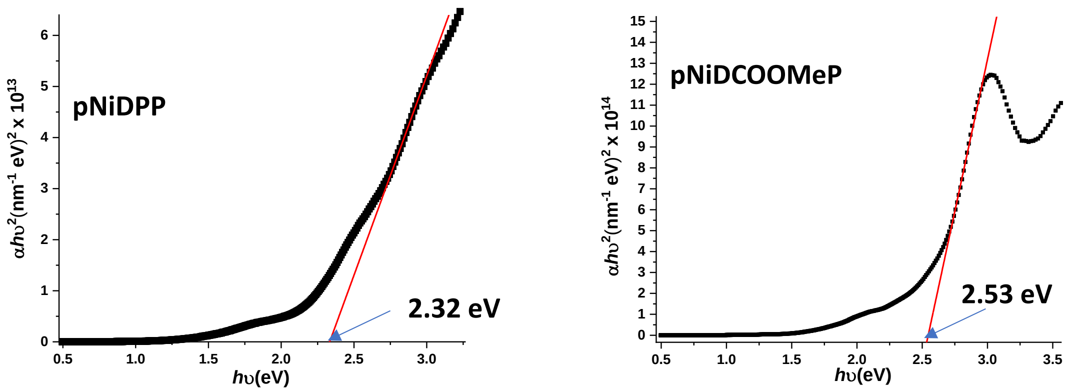

Fig. S11. Tauc's plots obtained from the UV-Vis-NIR absorbance of the oCVD coatings for energy band gap value estimation.

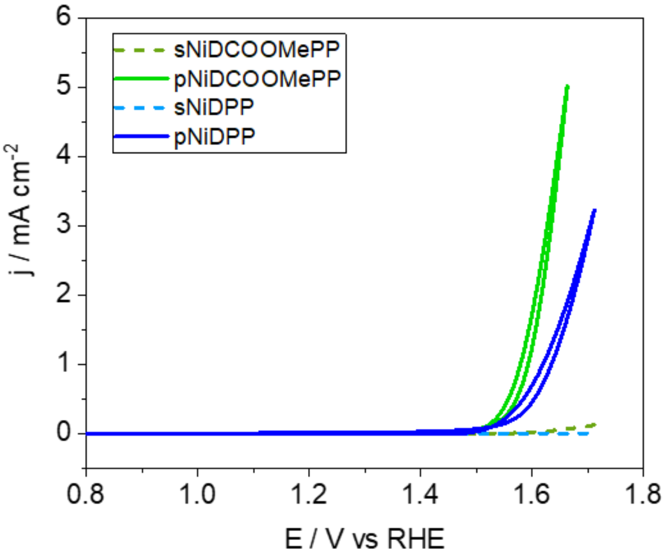

Fig. S12. Cyclic voltammetry recorded at 500 mV s^{-1}, in 1M KOH, of the oCVD and sublimed films from Ni(II) *5,15*-(di-4-methoxycarbonylphenyl)porphyrin (NiDCOOMePP) and Ni(II) *5,15*-(di-phenyl)porphyrin (NiDPP).

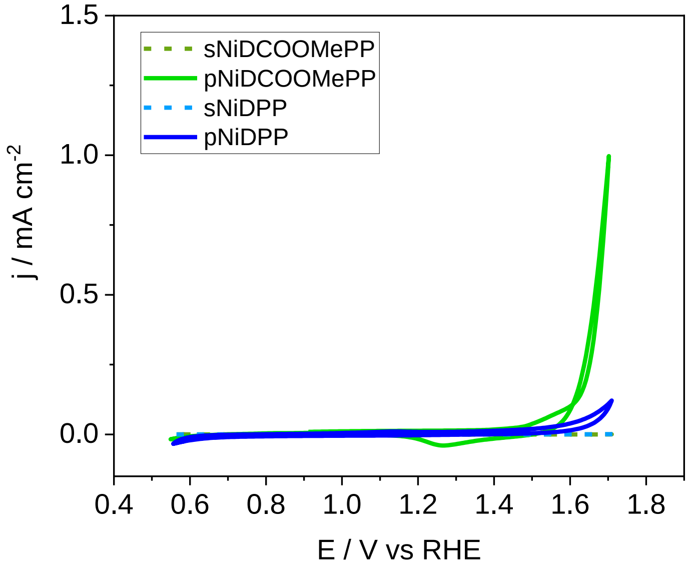

Fig. S13. Cyclic voltammetry recorded at 500 mV s^{-1}, in 0.5 M Na_2SO_4, of the oCVD and sublimed films from Ni(II) 5,15-(di-4-methoxycarbonylphenyl)porphyrin (NiDCOOMePP) and Ni(II) 5,15-(di-phenyl)porphyrin (NiDPP).

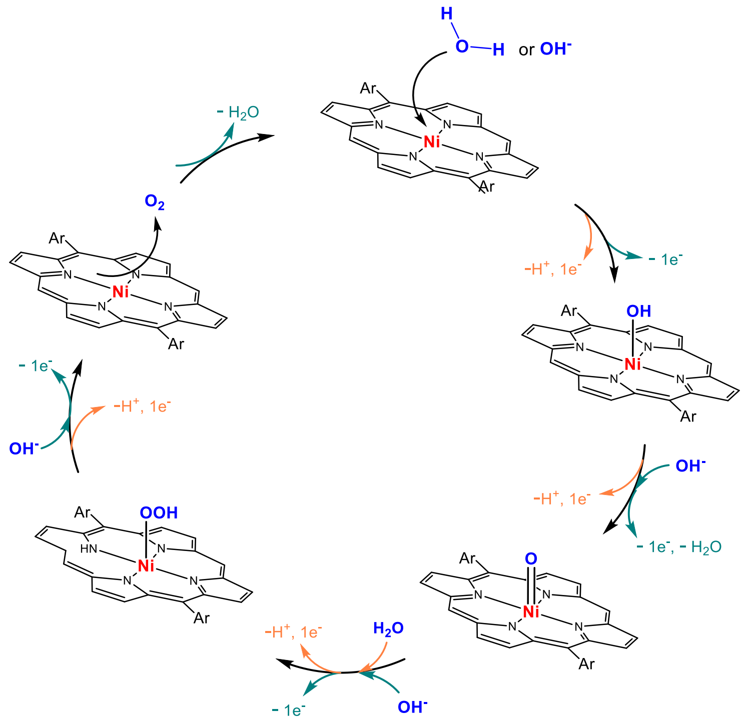

Fig. S14. Representation of the water nucleophilic attack pathway performed by the porphyrin monomers in acid (orange) and alkali conditions (green).

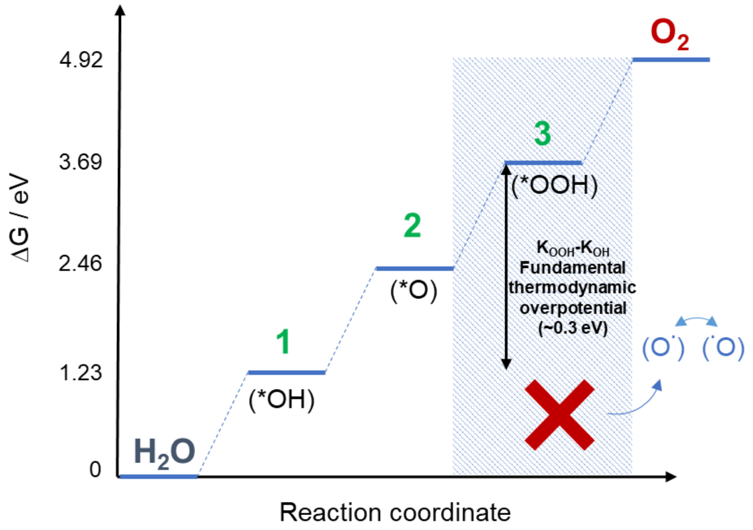

Fig. S15. Energy barrier diagram, considering optimal operation of a catalyst operating via a WNA mechanism. As represented in the diagram, catalyst operating with this mechanism have a minimum fundamental overpotential of 0.3 eV due to a scaling relationship between the intermediates (*OH) and (*OOH) resulting in a fixed energy difference of ~3.2 eV, instead of the expected 2.46 eV. Such intrinsic limitation is not existing on the ROC pathway, as no (*OOH) intermediate is formed, allowing the operation of the catalyst at lower overpotentials.

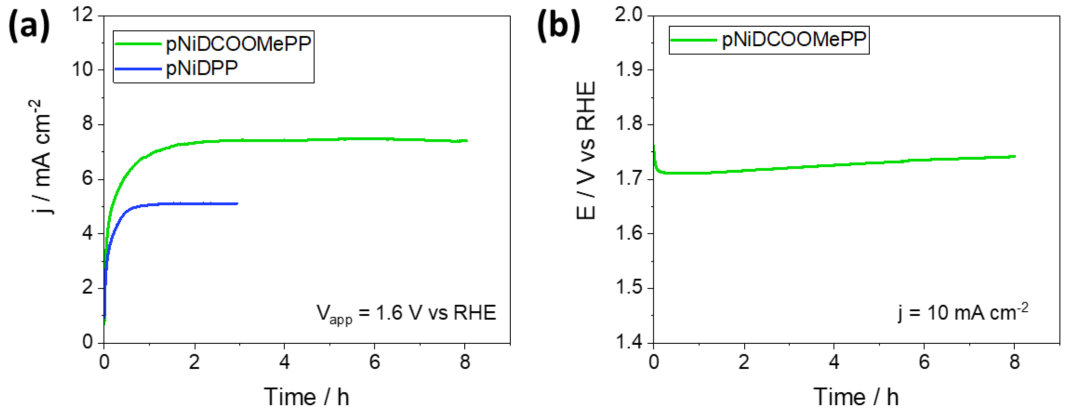

Fig. S16. Chronoamperometry measurement on the oCVD film, recorded at 1.6 V vs RHE applied potential in 1M KOH. (b) Potentiometry measurement on the pNiDCOOMePP film, recorded at fixed current density of 10 mA cm^{-2}.

Table S4. Relative elemental composition from XPS analysis of the oCVD thin films before (as-deposited) and after chronoamperometry.

Sample	Ni 2p %	O 1s %	N 1s %	C 1s %	Cl 2p %	Fe 3p %
**pNiDCOOMePP (as-deposited)**	1.0	17.6	4.2	71.3	2.5	3.4
**pNiDCOOMePP (after chronoamp.)**	1.3	15.3	4.4	76.8	1.2	1.0
**pNiDPP (as-deposited)**	1.6	11.4	6.9	76.7	1.1	2.4
**pNiDPP (after chronoamp.)**	2.0	21.8	5.1	66.1	1.2	3.8

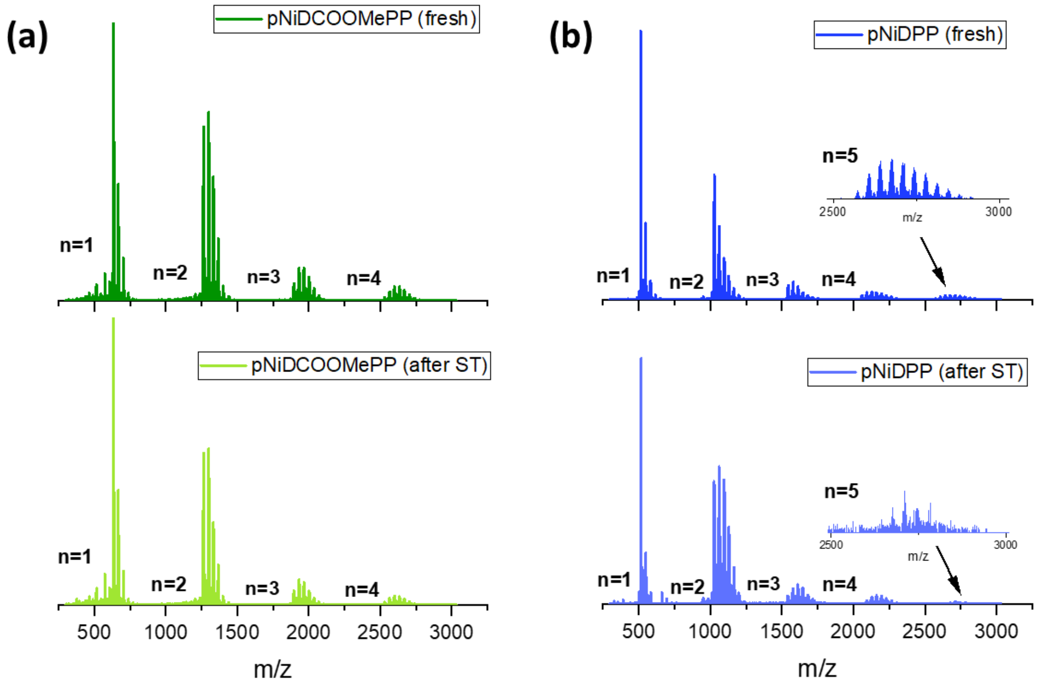

Fig. S17. Comparative LDI-HRMS spectra of **(a) pNiDCOOMePP** and **(b) pNiDPP** catalysts before (fresh) and after stability test (after ST), showing the presence of oligomers that indicates the retention of the catalyst on the electrode surface after operation.

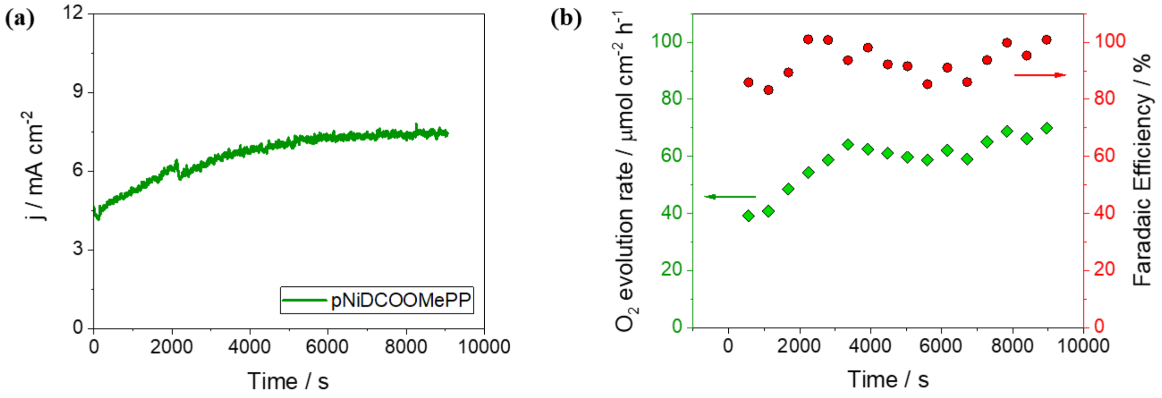

Fig. S18. (a) Chronoamperometry recorded during the gas evolving measurements on the pNiDCOOMePP film, performed at 1.6 V vs RHE applied potential in 1M KOH. **(b)** Calculated oxygen evolution rate and faradaic efficiency.

Table S5. Coordinates for triply fused NiDCOOMePP dimer.

	X	Y	Z
C	-1.67888445848896	-3.32812553374855	2.60569676497211
C	-1.09686266785950	-4.55934949614466	2.52871542638609
C	-0.18388914480262	-4.52304139254501	1.41248967075886
N	-0.22694333468474	-3.28387136842686	0.79530313831690
C	-1.15789510001967	-2.55183304208259	1.51354099310090
C	-1.47627161118895	0.74465369766982	-1.76444810717633
C	-1.99001286495007	0.48926618397539	-0.52665459456565
C	-1.29463272588759	-0.66017309256663	-0.01553340472364
N	-0.33223926282952	-1.08766780907088	-0.91376861817084
C	-0.41739512848756	-0.20977259713110	-1.98405470697475
C	-1.63153296626977	-1.30247956421463	1.16131904530630
C	3.61634937984843	-1.75617360643670	-3.74973703837379
C	2.64126173649424	-0.84259748118466	-4.08262532936591
C	1.57373544669083	-1.00519206183776	-3.13268597912412
N	1.87111719183899	-2.02003339476375	-2.22878618360473
C	3.10569226728130	-2.48567768838689	-2.61325412045493
C	0.45310415841335	-0.17849633148335	-3.07302923307482
C	2.80041210648958	-6.32697235641363	-0.02212799345095
C	3.71812619032472	-5.71857010865959	-0.84879102011058
C	3.16705072622412	-4.42328458185116	-1.16982911156804
N	1.96821278891849	-4.21378428896477	-0.53270524541575
C	1.74464013352317	-5.37658513117525	0.20136729204095
C	0.69680614440723	-5.55944212015271	1.10094638873417
C	3.77663704012683	-3.59529373930397	-2.10822240461538
Ni	0.81942278830611	-2.65061695831533	-0.71837507653376
C	0.24452655447920	0.81020116749208	-4.16203049694400
C	0.56066194811829	-6.85203572974013	1.82043123291797
C	-0.03227460807387	0.36865781989473	-5.46722039627268
C	-0.21430837198263	1.28155821507168	-6.50090917049576
C	-0.11884791329623	2.65863902357536	-6.25312607111908

C	0.16406504070367	3.10732430123751	-4.95406422376783
C	0.34363383079663	2.19050808937628	-3.92151548936245
C	-0.58301065880307	-7.64637464030241	1.63238909205489
C	-0.72242190382008	-8.85795404187953	2.30173223480817
C	0.27829649895697	-9.30076379330479	3.17879535352146
C	1.41910026311310	-8.50881990018993	3.37933350096492
C	1.55576322721811	-7.29742542909741	2.70664396164073
C	0.07757605556478	-10.60701333551363	3.86444803742804
C	-0.32150043078755	3.58917233560528	-7.39730372710679
O	1.12309309065105	-10.92475420138330	4.67746603069840
O	-0.21620782250066	4.89434479591120	-7.02137948525699
H	-2.41486484358301	-2.96950523856414	3.31987964573095
H	-1.23864580019802	-5.41583211231386	3.17904235469221
H	-1.77015290097408	1.51370077597335	-2.47027332378778
H	-2.79529693188983	1.00203549122674	-0.00810681309952
H	-2.38865266301566	-0.84377156911344	1.79633070718899
H	2.64955205652924	-0.10702291190370	-4.87986509456653
H	2.83870904878191	-7.32260580763139	0.40579152840748
H	-0.11045081978293	-0.70196882012609	-5.65939009801007
H	-0.43635464026356	0.94850147209041	-7.51475832744231
H	0.25075234455763	4.17543509005576	-4.76122646536616
H	0.58053301845007	2.53962604598201	-2.91595987635562
H	-1.35832556279040	-7.30597701328764	0.94510026165170
H	-1.60127369512511	-9.48585906706979	2.15520486204304
H	2.19190212765873	-8.84434323387596	4.06907945196534
H	2.43489846701489	-6.67426770870359	2.87340027062243
O	-0.90184795209923	-11.31927707526489	3.72845688484278
C	0.98688612610480	-12.18464599796825	5.36736339826223
H	1.89417264687507	-12.28519069987935	5.97118997875815
H	0.90507663867044	-13.00913983847593	4.64702126145757
H	0.09312204240653	-12.17899191367597	6.00494073159691
O	-0.55502713230682	3.24218335512388	-8.54194807856889

C	-0.40571352296143	5.84507628430732	-8.08967970837143
H	-0.29942060942795	6.83027168866103	-7.62490352602327
H	-1.40287455065427	5.72926939724394	-8.53418854676767
H	0.35084575907044	5.70018853867894	-8.87213490136790
C	4.97453362760739	-6.10515432056648	-1.42672773221461
C	5.75763098694165	-7.23672709884216	-1.38455642674460
C	6.91390593988724	-6.97565381670670	-2.19880989232287
N	6.84402386984789	-5.70369155127271	-2.75861749915493
C	5.65964770877528	-5.17999341957372	-2.29811196261488
C	5.67128476206683	-1.66862965448011	-5.33665177392300
C	4.90152197493738	-2.11007418777655	-4.28411849787387
C	5.63390137991062	-3.20197095841923	-3.68647943129257
N	6.83553116005000	-3.40944055169836	-4.31930263112453
C	6.87892139500346	-2.44907296436307	-5.32748397041462
C	5.07164776327381	-3.97677951106616	-2.67606794601836
C	11.39975345486290	-3.27658865949156	-6.08769425197729
C	10.41408744235697	-2.49565770416414	-6.61598062354477
C	9.18160267310629	-2.88617640999119	-5.97640276586717
N	9.40862272028616	-3.91901009974668	-5.08153846473802
C	10.76784353795742	-4.17327540084375	-5.15844386842035
C	7.97233511662472	-2.21548057558827	-6.15772500797132
C	10.41094397307695	-8.22024745790889	-2.87567838582018
C	11.39186714052354	-7.43839612068473	-3.41150592330662
C	10.76828628821255	-6.21037065917404	-3.82288235081988
N	9.42044051187150	-6.21735267296036	-3.50791869311726
C	9.19606090655514	-7.44335952756215	-2.89906034537991
C	7.99877376271360	-7.84362268537793	-2.30759716820195
C	11.41882721683004	-5.22035533401901	-4.53501301840204
Ni	8.12899285048901	-4.81252906788649	-3.91649980523734
C	7.88767288635607	-1.16448961972332	-7.20492407475313
C	7.88967500256829	-9.19283156836372	-1.69475340099777
C	7.96812651461498	-1.51421847475243	-8.56306378107930

C	7.88985326737540	-0.53824281202606	-9.55152385097103
C	7.73470235558091	0.81112767052538	-9.20263756672614
C	7.66302093501630	1.16921124228453	-7.84792746520299
C	7.73925590008115	0.18935695499314	-6.86145848376764
C	6.97623839103750	-10.12862266509930	-2.20890736897488
C	6.85227636390880	-11.38870742085586	-1.63251424692787
C	7.63720657590591	-11.73999424144818	-0.52468217480267
C	8.54732911762846	-10.80889812815838	-0.00168074664798
C	8.66982005971368	-9.54932800681521	-0.58249597735957
C	7.46021064401400	-13.10061670908695	0.05298014145487
C	7.65265412733977	1.81124565835231	-10.30267557288117
O	8.28559868182096	-13.31808933323609	1.11459994977686
O	7.48829549298086	3.07841803209640	-9.83181861836439
H	5.58617681224300	-8.15479670036349	-0.83282275511048
H	5.44910937666328	-0.88126785661470	-6.04851830973914
H	12.46254519009389	-3.27287985858954	-6.31295496714136
H	10.50092376830712	-1.70232171633669	-7.35069740149042
H	10.49087447306769	-9.22903776380075	-2.48587819049938
H	12.44261484181508	-7.67308248020972	-3.55695079051033
H	12.49121887473386	-5.32547795607544	-4.69566820943957
H	8.08176848675460	-2.56416664983860	-8.83537294286656
H	7.94081965684399	-0.80051783616132	-10.60829101373478
H	7.55414962626984	2.21733105283121	-7.57376057297058
H	7.69728562730849	0.46881015936670	-5.80817665477233
H	6.36711761534809	-9.85688618065216	-3.07174960200123
H	6.15123916131765	-12.12341460360355	-2.02861353535827
H	9.14985343864781	-11.07444074723542	0.86554356412431
H	9.36435320993806	-8.81980041371343	-0.16456072552344
O	6.67402523634729	-13.93440306476036	-0.36154393582608
C	8.15828121321993	-14.62266964780912	1.71758627719628
H	8.87708495433018	-14.63324700674734	2.54286381728928
H	8.39267840478742	-15.40774699607261	0.98681116357803

H	7.13637453426244	-14.77609256715759	2.08845940279540
O	7.71947702904453	1.54436025435884	-11.48989068171055
C	7.39164716843575	4.09208538869432	-10.85413465145324
H	7.27109069518785	5.03742616983451	-10.31593700826535
H	6.52702384011334	3.90032359661731	-11.50297081355557
H	8.30141000040191	4.10637331049167	-11.46846730179987

Table S6. Coordinates for doubly fused NiDCOOMePP dimer.

	X	Y	Z
C	-3.24797829359642	-4.55871887570245	-0.73727032918828
C	-2.26457192069462	-5.29224553429758	-0.13907171521767
C	-1.00608046220835	-4.79100907447097	-0.63589493829950
N	-1.22327721706482	-3.70730423119541	-1.47361408112019
C	-2.59551102503041	-3.55964668154267	-1.54068504049023
C	-2.32305120396583	0.74781727172542	-3.27897538113475
C	-3.28800517402613	-0.19557426324316	-3.07600933117134
C	-2.61527864165179	-1.40061061604740	-2.66858387148651
N	-1.24341125477708	-1.21288106816391	-2.67830490904247
C	-1.05168841339475	0.10618374562831	-3.04351809456228
C	-3.25670379692459	-2.52362234826011	-2.17930160942894
C	3.46641124850863	-0.58774099345811	-3.93567929701909
C	2.63229245950610	0.48215303585101	-3.77944174959929
C	1.36160382250884	-0.03547494416493	-3.34340082275313
N	1.45449097880053	-1.40679060026355	-3.14136489854809
C	2.73195360645098	-1.75707640770585	-3.53375540884180
C	0.18635518519996	0.71057814327610	-3.27299677785962
C	2.58316710175706	-5.74806358574348	-1.16316544493685
C	3.41383476501477	-5.13731230808191	-2.08285817252576
C	2.68782251221514	-3.97341455491125	-2.54993914209007
N	1.45831108729032	-3.88041155276149	-1.94370258739416
C	1.37681208995830	-4.97734288499499	-1.09757471119693
C	0.23444024819040	-5.38208634640785	-0.40112086728193
C	3.27554156544504	-3.04606302137787	-3.41326108051307
Ni	0.10723236069139	-2.55642526956541	-2.31388679851385
C	0.21845843615232	2.16768958113015	-3.56030337679792
C	0.35159414316950	-6.54268548167570	0.51699564085230
C	0.61853460995411	2.65434105956510	-4.81683338394889
C	0.63222598758635	4.02080874583268	-5.07582465721323
C	0.24484499753374	4.93355192687781	-4.08373760979299

C	-0.16271621247289	4.45577066981136	-2.82904800185749
C	-0.17732733587441	3.08687583979501	-2.57432591551303
C	-0.31665467698225	-7.75158438672771	0.25783149736631
C	-0.16941060715764	-8.83921009746860	1.11244582086522
C	0.65168268749100	-8.74474049897205	2.24556773714549
C	1.32574893126461	-7.54292184553301	2.50958945718900
C	1.17658317932395	-6.45663424512548	1.65159267175579
C	0.77422038711472	-9.94319730895515	3.12052195775291
C	0.28274244959075	6.38378212146489	-4.41737767686344
O	1.62462353228676	-9.73134489383451	4.16248254306871
O	-0.12876227739897	7.16021657733974	-3.37686251768519
H	-4.32438328318448	-4.64858947323598	-0.62056973655285
H	-2.37075865318585	-6.11073957873241	0.56445819217252
H	-2.44401210644125	1.78015898578406	-3.58965275663417
H	-4.36589719454980	-0.09114074035298	-3.16305285200389
H	-4.34597497135937	-2.53886927282446	-2.19006602803734
H	4.51172501554547	-0.57794967088560	-4.22062946082105
H	2.85272052523131	1.53109511480167	-3.94452397766862
H	2.73415723282949	-6.68201301322360	-0.63584169927139
H	0.90298039083211	1.94579603690939	-5.59520725550128
H	0.93347071110820	4.40696396173358	-6.04964126491360
H	-0.46217544023408	5.16183183461452	-2.05618617118029
H	-0.48872538005566	2.71499401749641	-1.59750961630356
H	-0.93509955131955	-7.83740752931248	-0.63602736555142
H	-0.67536149910776	-9.78413206613109	0.91403068911937
H	1.96220068443321	-7.46527722111035	3.38961994873835
H	1.69697182220776	-5.52044988513162	1.85725368632730
O	0.19125449127290	-10.99785360533286	2.93831133242853
C	1.78710445584538	-10.86039425100478	5.04654332856480
H	2.49729223300595	-10.53124477514830	5.81138318215185
H	2.18016970376967	-11.72494375571752	4.49597337568841
H	0.82528694168175	-11.13413532353074	5.49966721620136

O	0.63792761152568	6.84070167794934	-5.49013972872599
C	-0.11359834550746	8.57837179296029	-3.64149074361455
H	-0.46985330356176	9.05014541480155	-2.72042751855704
H	-0.77552080183937	8.81890314189785	-4.48371002973116
H	0.90392536619314	8.91335523055886	-3.88208826409442
C	5.50087361297750	-6.86239773500326	-0.64199818031101
C	6.60417687466725	-7.63201647980548	-0.40581301450051
C	7.33767598756943	-7.70682778217432	-1.64239196673215
N	6.73070600401568	-6.90396579469575	-2.59982051207840
C	5.58819146013871	-6.40673727527608	-2.00336431549405
C	5.27559418523791	-2.92405438609649	-5.05941168950313
C	4.50528678957123	-3.46859854705829	-4.04983116833182
C	5.16843596568326	-4.70136238674130	-3.67606599586363
N	6.29672161335418	-4.90532252888812	-4.43317805976710
C	6.36489318705077	-3.82379982561096	-5.29976681228396
C	4.72732910437575	-5.46796283174851	-2.59476061834135
C	9.33535090913486	-6.05732884748912	-8.10663015366898
C	8.83765866758841	-4.79171914596712	-7.98865028082002
C	8.06839237357997	-4.75529865788838	-6.76856390846307
N	8.16312733943476	-5.97235974547236	-6.11092877771352
C	8.93910343821070	-6.77541705391847	-6.92458469756945
C	7.25698580866039	-3.69443453103254	-6.36851848212817
C	9.80561596424276	-9.83709086049488	-3.53075898497029
C	9.98953345689308	-9.75444104007439	-4.88066240293176
C	9.28199518669577	-8.58387844512843	-5.32820562362928
N	8.61994921615237	-7.98827221266598	-4.26804704633735
C	8.94002317315503	-8.74419604643011	-3.15659951171967
C	8.41240451838548	-8.56377096083621	-1.87559163310206
C	9.39225785317697	-8.04272780106394	-6.59624085869888
Ni	7.44861122525129	-6.44613026105786	-4.35938686693677
C	7.24101462443741	-2.42588494013813	-7.13895910062919
C	8.95192496603722	-9.39181835694491	-0.76697291753036

C	6.83882168788774	-2.38689906000954	-8.48504421525569
C	6.80106621339566	-1.18143439943433	-9.17834911865829
C	7.16258036914177	0.01458880381971	-8.54129703443525
C	7.56174348301998	-0.01478086081426	-7.19654888036172
C	7.59725528740075	-1.22275569122808	-6.50557117582771
C	8.15604258761337	-10.34626032815297	-0.11029237358769
C	8.68033689135279	-11.11689920036154	0.92222117881333
C	10.01463716053701	-10.95218632512534	1.32206117372772
C	10.81858777642425	-10.00802951200680	0.66558342053105
C	10.29082046171125	-9.24011648380830	-0.36900997218971
C	10.51883569886637	-11.80028759997949	2.43663424593817
C	7.09738085486931	1.27415769826288	-9.33251077354907
O	11.82768964542778	-11.55106206934130	2.71879367599964
O	7.44602182698404	2.36235944219438	-8.59224107163756
H	4.73753940359631	-6.56320892474452	0.06646897925630
H	6.89903086166921	-8.10940478650247	0.52223426024781
H	5.08336034565846	-2.03695727225629	-5.65051395606227
H	9.95465653759705	-6.46878514606356	-8.89880874551982
H	8.96091749710039	-3.95501936653018	-8.66749677803675
H	10.18930146931841	-10.58263421757940	-2.84236771487423
H	10.57459824363440	-10.40485304488580	-5.52488331210658
H	9.97211803776665	-8.58892786471355	-7.33943383002208
H	6.53180645149829	-3.31051511923556	-8.97646459074708
H	6.48036032578460	-1.14031908740118	-10.21934551883974
H	7.84608349176905	0.91138130414076	-6.69958725925451
H	7.91080502170645	-1.24761610203764	-5.46125927138405
H	7.12378588220169	-10.48980699231992	-0.43079969485757
H	8.07212397189165	-11.86409414329551	1.43207423240781
H	11.85445292484053	-9.87630519366434	0.97407989185426
H	10.91314004451723	-8.50119882025884	-0.87517656442631
O	9.85236921487980	-12.62420694483385	3.03893942150843
C	12.37236530955286	-12.34237147251005	3.79521913757428

H	13.41419579902677	-12.02189052944474	3.89379647421974
H	12.31497350598078	-13.41160651592510	3.55270358351852
H	11.81849007559788	-12.15856578194815	4.72516227294201
O	6.77144804067846	1.34100155188292	-10.50498004777991
C	7.40009307081708	3.61585868641677	-9.30587283795169
H	7.70192587715362	4.37588742825821	-8.57855865870109
H	6.38424504847232	3.81068495870612	-9.67369402049171
H	8.09047358205939	3.59696161695593	-10.15926456026323

Table S7. Coordinates for triply fused NiDPP dimer with intramolecular cyclization.

	X	Y	Z
C	-2.06120832220257	-4.51326952335146	2.16063187832075
C	-1.06833809134202	-5.45994080747624	2.21338709082902
C	-0.08215385728185	-5.04243080633935	1.25559870127964
N	-0.40861698110820	-3.89600352625629	0.61688721847371
C	-1.64149201150642	-3.54116800411975	1.16180994443789
C	-1.85407156708855	0.28328382585220	-1.53857617455271
C	-2.59699102945073	-0.34925244182008	-0.58185483361216
C	-1.86892195876838	-1.52413028794115	-0.18853162830470
N	-0.68232743195074	-1.62185596498076	-0.89574926809724
C	-0.66934237898242	-0.50143370350036	-1.73484235631178
C	-2.33119326308154	-2.41298341603128	0.77515818544769
C	3.43745771629895	-1.68895594942991	-3.56687365446959
C	2.44218294682895	-0.72010686701942	-3.62845098532164
C	1.45192289987461	-1.12531590886692	-2.67134197125072
N	1.75884055553011	-2.27458910378150	-2.01875208898329
C	2.97454913490950	-2.63749819947959	-2.55317415984372
C	0.38002468055602	-0.24928821077980	-2.61246198760969
C	3.22827999874425	-6.45597450729727	0.12840882422901
C	3.97924052350086	-5.81983984580647	-0.84267750303637
C	3.22317756126044	-4.64249947996650	-1.22022693610475
N	2.04931317753655	-4.54808140684737	-0.51509889868391
C	2.04825233236764	-5.67578885945522	0.32628693603752
C	0.99607669982562	-5.91894590285737	1.20062288954864
C	3.69848880178413	-3.75898398371146	-2.19462755386926
Ni	0.67600532512240	-3.08418365133059	-0.70192929751767
C	0.68393792613053	0.81712780166488	-3.60323714582451
C	0.70017051651939	-6.98550605820970	2.18951471966846
C	1.95362191295685	0.51839432231775	-4.22212245597326
C	2.47830884512429	1.36357258004624	-5.19262996003879
C	1.75883694264383	2.51217703020577	-5.56531475203687

C	0.53276877067059	2.80261139420708	-4.96951789054375
C	-0.01173221227573	1.95652182252851	-3.98455301322040
C	-0.56792263717698	-6.69956403077222	2.80855299084604
C	-1.08640884222256	-7.54621501352075	3.77933084416765
C	-0.35620945800487	-8.68812644498929	4.15147229803429
C	0.87264894306220	-8.96880676675706	3.55492768054832
C	1.40837988093012	-8.11975674676464	2.57066047392660
H	-2.99124552129192	-4.44293167933422	2.71861244378200
H	-2.08863911650865	1.20144094409747	-2.06553620355466
H	-3.55802029352097	-0.05698368669557	-0.16762837659974
H	-3.29039964201575	-2.19816906376260	1.24517655083514
H	3.46664160908136	-7.37425155161245	0.65263732588173
H	3.43784227761032	1.14022873873865	-5.65973108136210
H	-0.01518031195724	3.69709853544239	-5.26765696605404
H	-0.97106262153918	2.20849892253950	-3.53461618118020
H	-2.04744021273756	-7.32893590130740	4.24739576273848
H	1.42812011839225	-9.85839764102890	3.85392235408892
H	2.37029453394802	-8.36245347949218	2.12130237476886
C	5.23539864241847	-6.08172009956227	-1.47988807216465
C	6.23107030448216	-7.05014529643030	-1.41786052649010
C	7.22191056469440	-6.64412961174201	-2.37391871109820
N	6.91505757216681	-5.49481605069757	-3.02640388310958
C	5.69862897909225	-5.13275274149220	-2.49314298358405
C	5.44287038566236	-1.31643291661596	-5.17774533308220
C	4.69332172407817	-1.95112785684340	-4.20462007026544
C	5.45006056875482	-3.12775239755360	-3.82619350833835
N	6.62303104776073	-3.22307223403724	-4.53267937358341
C	6.62276533228807	-2.09676269992644	-5.37596236376211
C	4.97499167527388	-4.01097834308927	-2.85139156402698
C	10.72972839410489	-3.26205616943309	-7.21431228270501
C	9.73581029321530	-2.31653696695521	-7.26836079072500
C	8.75150724710008	-2.73197580288587	-6.30784316703098

N	9.07991595309151	-3.87626019445283	-5.66639694363094
C	10.31243171857784	-4.23152681351195	-6.21187862586751
C	7.67363200234502	-1.85501842234128	-6.25227774593829
C	10.53477316930064	-8.04481375732124	-3.49642100762731
C	11.27632157967322	-7.41380175203535	-4.45519324555416
C	10.54498875448972	-6.24261885452262	-4.85348963332339
N	9.35769075467539	-6.14571761599107	-4.14732554574003
C	9.34726438384084	-7.26325206325659	-3.30435363647147
C	8.29684261116806	-7.51668032165032	-2.42833116071512
C	11.00465160321054	-5.35676581043270	-5.82118674820235
Ni	7.99718533628055	-4.68618402862866	-4.34466310373724
C	7.96812458185610	-0.79008466126862	-7.24334342344203
C	7.99423382075628	-8.58193024559572	-1.43598960372216
C	9.23477898423842	-1.07781098156256	-7.86474602019518
C	9.75191509817328	-0.23235322179114	-8.83729017181592
C	9.02211180820313	0.91008542257870	-9.20866871955003
C	7.79501178988620	1.19272490766737	-8.60945465069722
C	7.26051800747901	0.34489253098006	-7.62337819986528
C	6.72229703984258	-8.28579882976104	-0.82033214972413
C	6.19797330869909	-9.13022095895296	0.15088941539545
C	6.92035308243979	-10.27573860591105	0.52787965016329
C	8.14848281134114	-10.56363639982325	-0.06457485165500
C	8.69254809161606	-9.71825660287635	-1.05037891763910
H	5.20250705366086	-0.40012347556742	-5.70450036890818
H	11.65882120547253	-3.33341888641772	-7.77371961665734
H	10.77158079175397	-8.96031182428536	-2.96585116757336
H	12.23846300917226	-7.70474971832480	-4.86776824357801
H	11.96410634198361	-5.57136466218668	-6.29080498242507
H	10.71164056532663	-0.45109033068457	-9.30737639415046
H	7.24003648333399	2.08289469339314	-8.90764595360249
H	5.23683168111078	-8.90877392207479	0.61556863253323
H	8.69868421296473	-11.45563024070688	0.23690282715541

H	9.65376591643227	-9.96808986839694	-1.49748014381136
H	9.41987805933391	1.58016509423523	-9.97158611234952
H	2.16428647552182	3.17994281495786	-6.32611747055479
H	6.51534561530501	-10.94298518429929	1.28936880895805
H	-0.75514811175583	-9.35903841169484	4.91303692986476
H	6.30021480891051	0.58956289483234	-7.17168323931190

Table S8. Coordinates for doubly fused NiDPP dimer with intramolecular cyclization.

	X	Y	Z
C	-3.32696033410742	-3.88178270288835	-0.80885086886319
C	-2.47020946926771	-4.89420416400502	-0.47726203695656
C	-1.16078226394359	-4.49266693523108	-0.90697188570390
N	-1.21531210072590	-3.23070104236750	-1.50449671567295
C	-2.54290008986979	-2.84844393909552	-1.42831181285188
C	-1.59014842704573	1.47106325445472	-2.93399570590261
C	-2.71957698637498	0.78420783713571	-2.56000534254896
C	-2.29218290695927	-0.58193503934789	-2.28656079733762
N	-0.92485642506968	-0.72018150043295	-2.51825819823130
C	-0.52454713458247	0.50823584882652	-2.90472563941433
C	-3.06793646418028	-1.61570381454449	-1.80824507589306
C	3.78260183406001	-0.59577252882029	-4.16729396764450
C	3.06877594118800	0.56571590253225	-4.05773011402561
C	1.78855753194489	0.22142504350733	-3.51161813523625
N	1.73555776892669	-1.15315737879617	-3.24172846189215
C	2.94851667619396	-1.65518119078755	-3.67238620269794
C	0.68510294890734	1.04502588975190	-3.33445521642130
C	2.26781082433127	-5.66512407401439	-1.35997166173876
C	3.28978206139925	-5.08335785383754	-2.11374315336778
C	2.72769755895560	-3.82321046407980	-2.61199045272069
N	1.46598777347890	-3.60195843058262	-2.11156206767841
C	1.19001431979252	-4.71935046614384	-1.39158936944309
C	0.02659109276713	-5.21255653156971	-0.81644851892943
C	3.39175819464238	-2.98312037074616	-3.50688757146439
Ni	0.25596390299430	-2.18331349418816	-2.35429839680755
C	0.40728371879352	2.47764750010103	-3.61440145205576
C	0.36910230492727	-6.57332450117306	-0.33294839660171
C	1.22962767717635	3.51594647369225	-4.03722357405894
C	0.68121981960181	4.79704612304957	-4.22446936633921
C	-0.67295770462675	5.03805577018084	-3.99339849275239

C	-1.51914589623745	4.00169646041442	-3.56350232532304
C	-0.98970755312315	2.73210873182216	-3.37246825625499
C	-0.39192746163376	-7.53731076880106	0.31533649623130
C	0.17144732292580	-8.79740452094138	0.58687435224850
C	1.47669103674078	-9.08985349972406	0.19775274673174
C	2.26816693806097	-8.12160779197929	-0.44372870501077
C	1.73717765139239	-6.85925603718572	-0.69117687355768
H	-4.39850537795387	-3.82088331222179	-0.63931054505024
H	-2.70721661395340	-5.83394328424055	0.00828942894572
H	-3.74504217429543	1.12958618247587	-2.45781485542786
H	-4.13543635903783	-1.45122190520502	-1.66538962555858
H	4.80578901295995	-0.72358727958714	-4.50174618763492
H	3.38986517151861	1.56733871189128	-4.32056480946008
H	2.29126920290987	3.35574287943329	-4.22008187663151
H	1.32689965092777	5.61148238392440	-4.55488070735551
H	-2.57759388427609	4.19267721785034	-3.38164424174892
H	-1.41979731715970	-7.33540743012617	0.61309312476335
H	-0.42765308023249	-9.55397072452291	1.09480553141103
H	3.28615440483302	-8.36225976493260	-0.74629199895825
C	5.18898034126087	-6.79384556968273	-0.44776453507475
C	6.23815085814421	-7.58425306308462	-0.06666646329600
C	7.12529001579900	-7.67386676473572	-1.18946273949706
N	6.63905446261105	-6.89561125582913	-2.24900001453657
C	5.43709089526548	-6.38159809756305	-1.80111606069319
C	5.32776921506507	-3.24527789532887	-5.27166353200942
C	4.56144550770445	-3.54419329955351	-4.14262304755976
C	5.17004588152787	-4.75292934363413	-3.57591126936497
N	6.30209329715128	-5.11736886007525	-4.26634333840884
C	6.36969502222977	-4.23047569889821	-5.29185706153606
C	4.63499962239365	-5.45039036128530	-2.49202187845163
C	9.62808227116682	-6.45825970236590	-7.71335341784311
C	8.87527715717393	-5.33145819589627	-7.89431983377446

C	8.08283654303419	-5.16219813325145	-6.70946824257687
N	8.34996251371749	-6.18873946191422	-5.80010457002389
C	9.31216835219197	-6.97440250021205	-6.40948344324882
C	7.13718107941107	-4.17559073636591	-6.44749496039891
C	9.97762698882846	-9.50600516348622	-2.53864923667323
C	10.49135286359203	-9.49951410446140	-3.81280232269576
C	9.75120924988421	-8.47901589208349	-4.54396157722164
N	8.78614732746959	-7.89629977216520	-3.72457864658041
C	8.93296549612933	-8.52003975749474	-2.53803141839984
C	8.26036210001713	-8.46249239219720	-1.32128621168255
C	9.96663776621300	-8.06417325857880	-5.84039243125728
Ni	7.51428788707835	-6.53353710738974	-4.02071243674671
C	6.58846838982929	-3.04333882441228	-7.23464549170815
C	8.94829517138539	-9.44189560624431	-0.44057033115752
C	6.95095808306895	-2.52307159734187	-8.47016773611570
C	6.19161303225223	-1.48092681920745	-9.03241943878463
C	5.07385723853475	-0.98016030819751	-8.36859193716051
C	4.70164534837448	-1.48212428528508	-7.10958977120455
C	5.46095144398693	-2.49080348406056	-6.52402539837708
C	8.74503985671374	-9.79530564819455	0.88860236212304
C	9.56466509488972	-10.77616365461617	1.47430110354350
C	10.57506817661148	-11.39749630167700	0.74060857480103
C	10.79744836057637	-11.05126401890302	-0.60311991956559
C	9.99619618569973	-10.08250445250799	-1.19315264603690
H	4.34455650082498	-6.46883189466262	0.14930523673662
H	6.39456752138683	-8.05778467954130	0.89601654672949
H	10.35123550427350	-6.90368451832485	-8.39106399350621
H	8.85181432566034	-4.67874887853711	-8.75939386088345
H	11.28904385388372	-10.09630999823877	-4.24732834310235
H	10.72278502395964	-8.57226764925789	-6.43796433092108
H	7.81108169426357	-2.91279919123521	-9.01244209763536
H	6.47614771035818	-1.07349174459360	-10.00315314762185

H	3.82356720941054	-1.08584736502634	-6.60197082175874
H	7.96712287619400	-9.32132677310603	1.48554419572447
H	9.40547372461559	-11.05231015564002	2.51726335473443
H	11.58987775819342	-11.53672003120220	-1.17432131406194
H	-1.07904295813098	6.03864706889858	-4.14574845776397
H	4.47895531058003	-0.18886083388994	-8.82613498559676
H	1.89347690935075	-10.07869973146306	0.39229983194858
H	11.19918162049795	-12.15697170492137	1.21282948506672

References

[1] G. Bengasi, K. Baba, G. Frache, J. Desport, P. Gratia, K. Heinze, N. D. Boscher, *Angewandte Chemie International Edition* **2019**, *58*, 2103-2108; bG. Bengasi, K. Baba, O. Back, G. Frache, K. Heinze, N. D. Boscher, *Chemistry – A European Journal* **2019**, *25*, 8313-8320; cK. Baba, G. Bengasi, D. El Assad, P. Grysan, E. Lentzen, K. Heinze, G. Frache, N. D. Boscher, *European Journal of Organic Chemistry* **2019**, *2019*, 2368-2375.

[2] G. Bengasi, L. Quétu, K. Baba, A. Ost, J. P. Cosas Fernandes, P. Grysan, K. Heinze, N. D. Boscher, *European Journal of Inorganic Chemistry* **2020**, *2020*, 1938-1945.

[3] F. Neese, WIREs Comput. Mol. Sci. 2012, 2, 73–78.

[4] F. Neese, WIREs Comput. Mol. Sci. 2018, 8, e1327.

[5] F. Neese, F. Wennmohs, U. Becker, C. Riplinger, J. Chem. Phys. 2020, 152, 224108.

[6] A. D. Becke, Phys. Rev. A 1988, 38, 3098–3100.

[7] J. P. Perdew, Phys. Rev. B 1986, 33, 8822–8824.

[8] F. Weigend, R. Ahlrichs, Phys. Chem. Chem. Phys. 2005, 7, 3297–3305.

[9] A. Schäfer, H. Horn, R. Ahlrichs, J. Chem. Phys. 1992, 97, 2571–2577.

[10] A. Schäfer, C. Huber, R. Ahlrichs, J. Chem. Phys. 1994, 100, 5829–5835.

[11] F. Weigend, Phys. Chem. Chem. Phys. 2006, 8, 1057–1065.

[12] S. Grimme, S. Ehrlich, L. Goerigk, J. Comput. Chem. 2011, 32, 1456–1465.

[13] S. Grimme, J. Antony, S. Ehrlich, H. Krieg, J. Chem. Phys. 2010, 132, 154104.

[14] F. Neese, F. Wennmohs, A. Hansen, U. Becker, Chem. Phys. 2009, 356, 98–109.

[15] R. Izsák, F. Neese, J. Chem. Phys. 2011, 135, 144105.
